# Recent Progress in the Synthesis, Functionalization, and Biological Outlook of Pyrimidines

**DOI:** 10.1021/acsomega.5c04880

**Published:** 2025-10-06

**Authors:** Glanish Jude Martis, Praveen S. Mugali, Santosh L. Gaonkar

**Affiliations:** † Department of Chemistry, 125853 Manipal Institute of Technology, Manipal Academy of Higher Education, Manipal, Karnataka 576104, India; ‡ Department of P. G. Studies in Chemistry, Alva’s College (Autonomous), Dakshina Kannada, Moodubidire, Karnataka 574227, India

## Abstract

Pyrimidine-containing heterocyclic molecules are highly important in medicinal chemistry because of their versatile biological activities and their potential for structural modification. These scaffolds have consistently attracted the attention of chemists and biologists alike and serve as key building blocks in the design and development of bioactive compounds. Recent advances in pyrimidine chemistry—including novel synthetic methodologies, named reactions, catalytic strategies, and functionalization techniques—have further expanded their applicability. The biological importance of pyrimidine derivatives has been instrumental in the development of several commercially available drugs in recent years. Given the ongoing global research in this area, there is a clear need to review and highlight recent developments in a systematic manner. This review not only provides insights into current trends but also serves as a valuable resource for researchers in the pharmaceutical industry and academic institutions engaged in early-stage drug discovery efforts.

## Graphical abstract

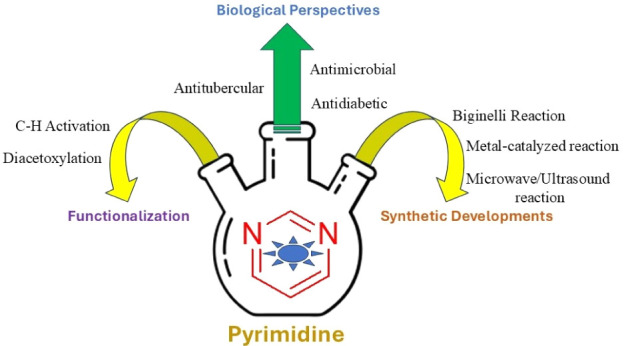

## 1 Introduction

Owing to their versatile behavior and nature, heterocyclic molecules are widely attractive across all fields of chemistry, which makes them special.^1^ Likewise, pyrimidine has been a core part of genetic research since the 1930s. The most profound genetic materials, namely DNA and RNA, contain purines and pyrimidines as nitrogenous bases (Figure 1).^2–6^ Among these, thymine, uracil, and cytosine have pyrimidine cores with several biologically important features, highlighting their interest in the fields of chemical biology and medicinal chemistry.^7–11^

However, synthetic organic chemistry has been a boon for the development of all the drugs that are commercially available today in global pharmacies.^12,13^ Similarly, pyrimidine chemistry has furnished groundbreaking results that have aided in drug design and development.^14–17^ One of the familiar and popular reactions proposed by Pietro Biginelli has emerged as an effective method with several advantages, such as diverse reaction optimizations. The very important factor is their modifications and improvements, which lead to the production of new lead molecules.^18–20^ Pyrimidine–-tetrahydropyrimidinethione hybrids produced via the Biginelli reaction have been used as an effective antimycobacterial agents with promising results.^21^

**Figure 1:**
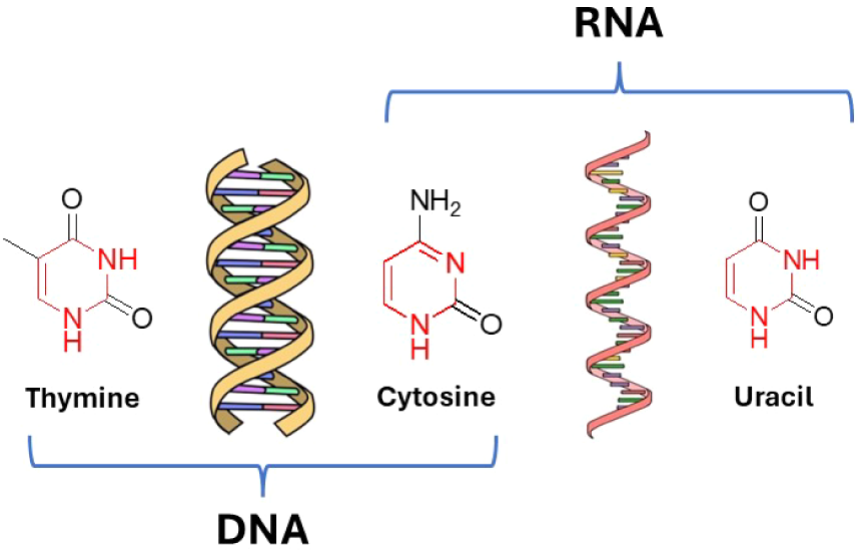
Pyrimidines in DNA and RNA.

**Figure 2:**
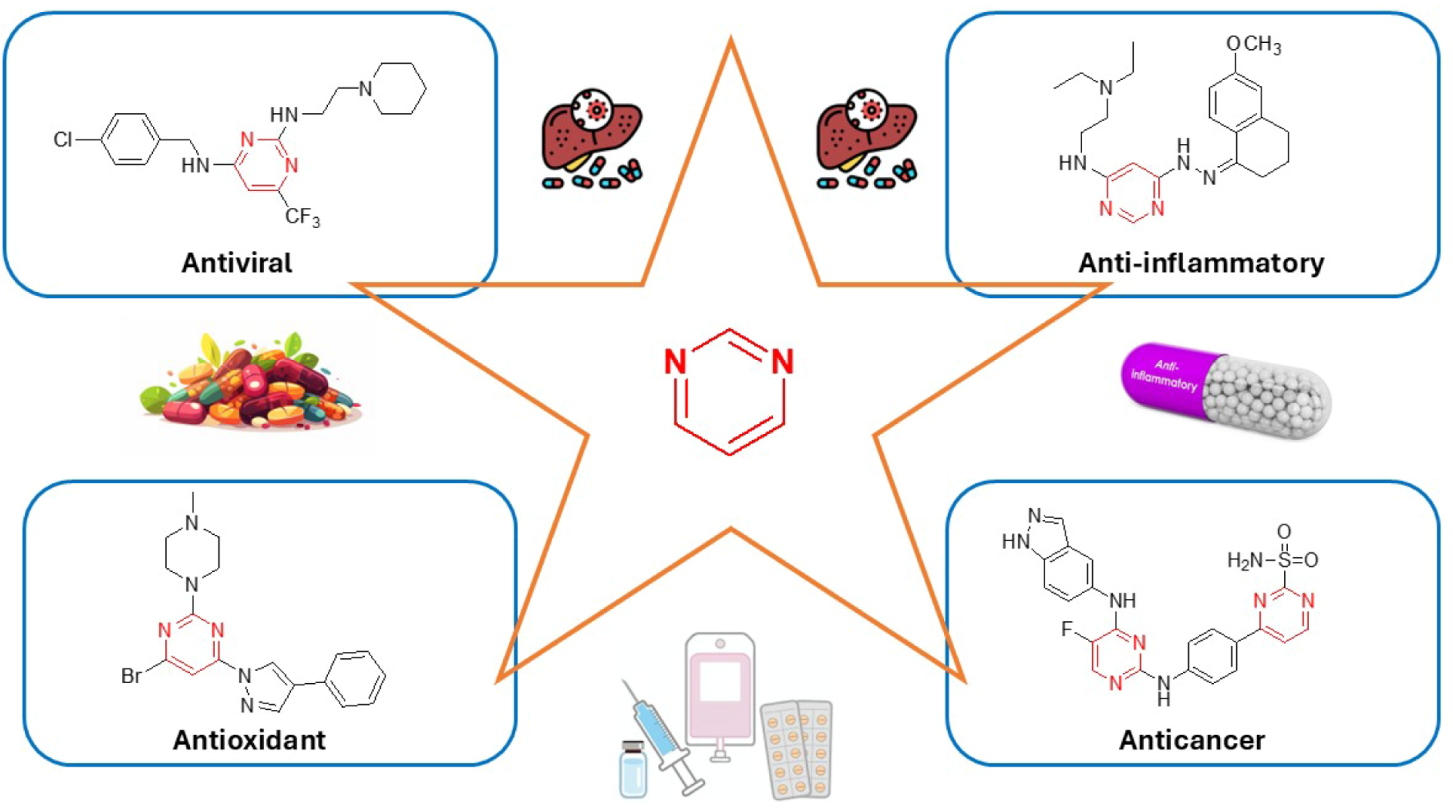
Recently developed pyrimidine-containing biologically active agents.

**Scheme 1:**
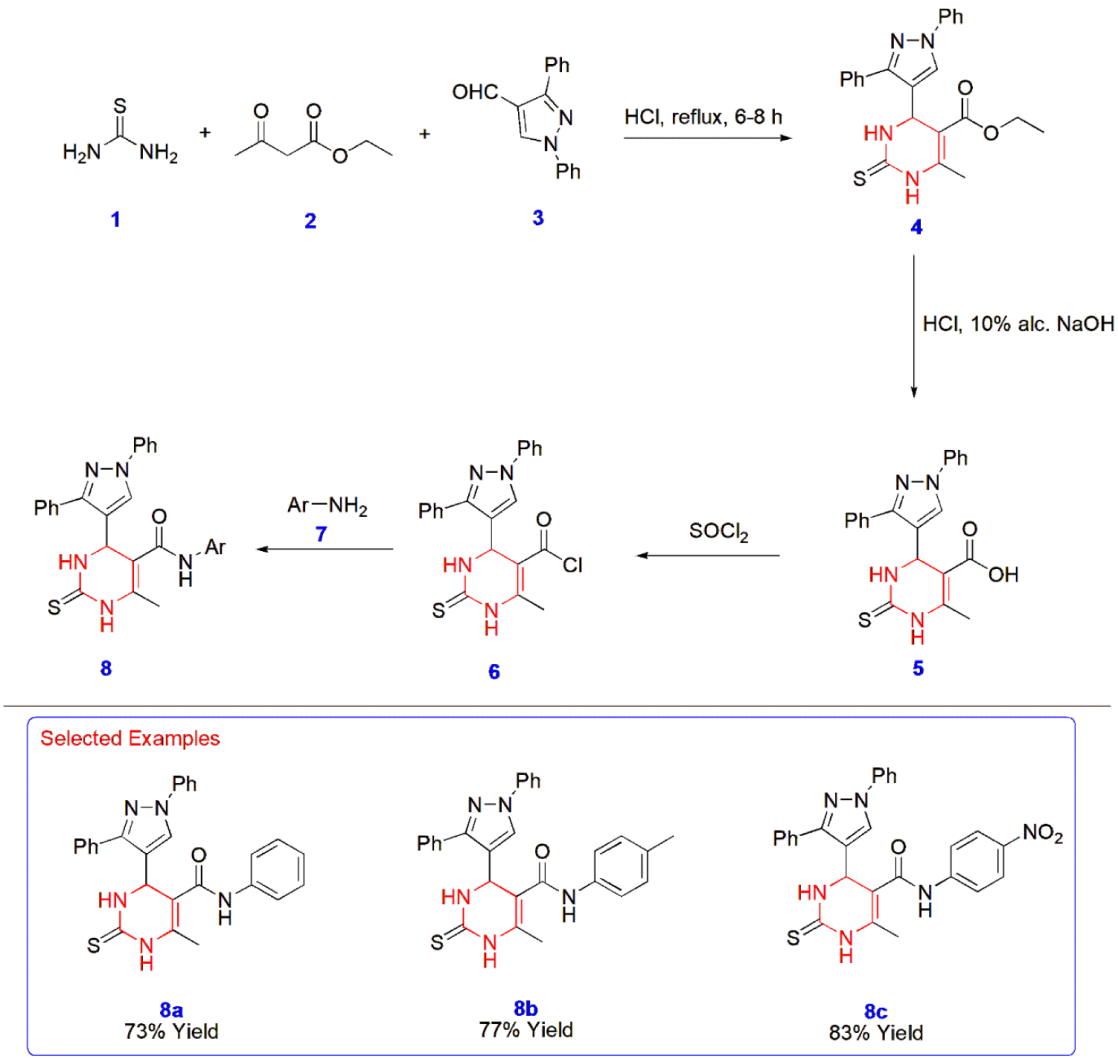
Synthesis of Pyrimidine–Pyrazole Hybrids **8** via the Biginelli Reaction

**Scheme 2:**
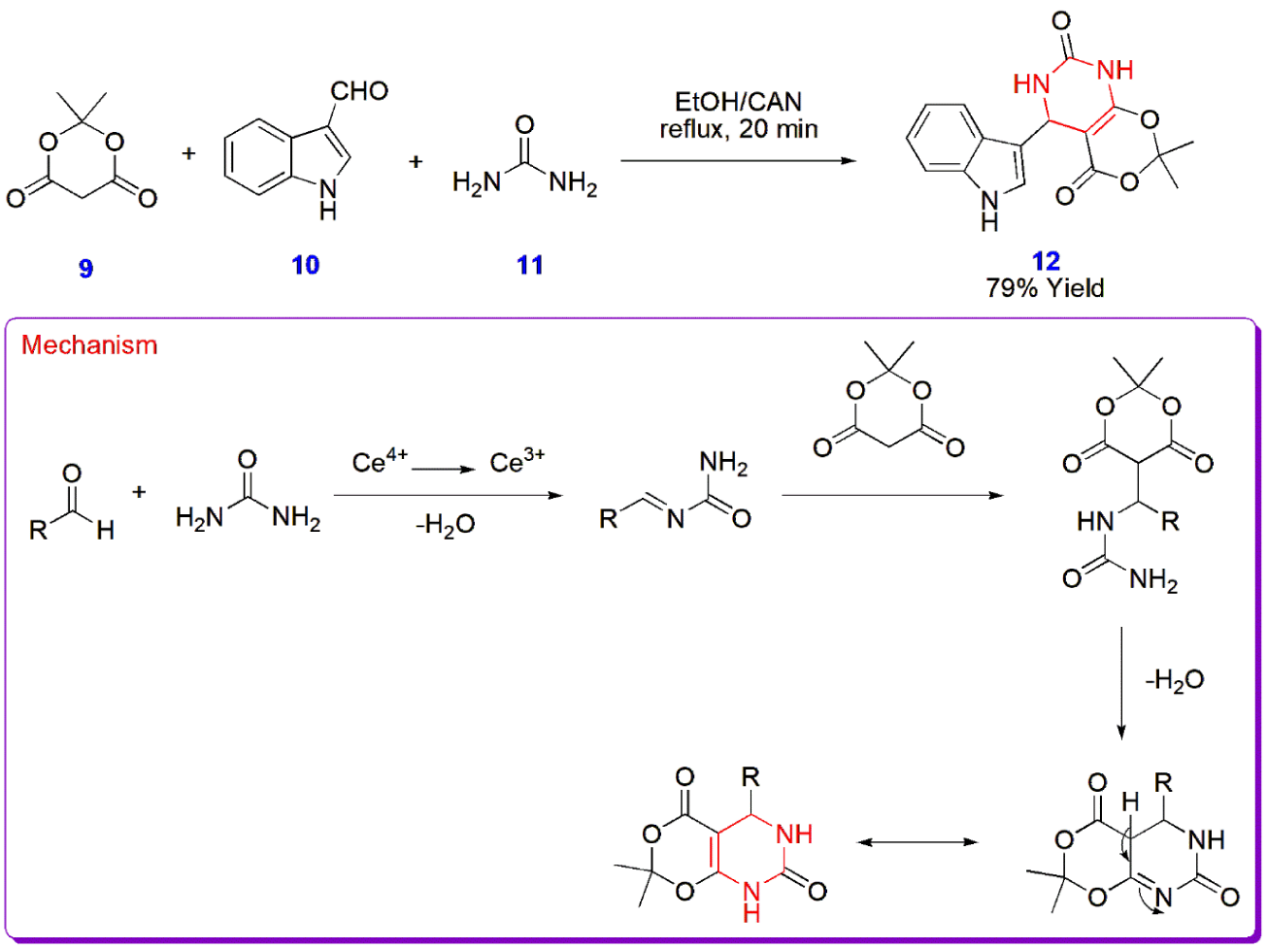
Synthesis of [1,3]­Dioxino­[4,5-*d*]­pyrimidine Derivative **12**

**Scheme 3:**
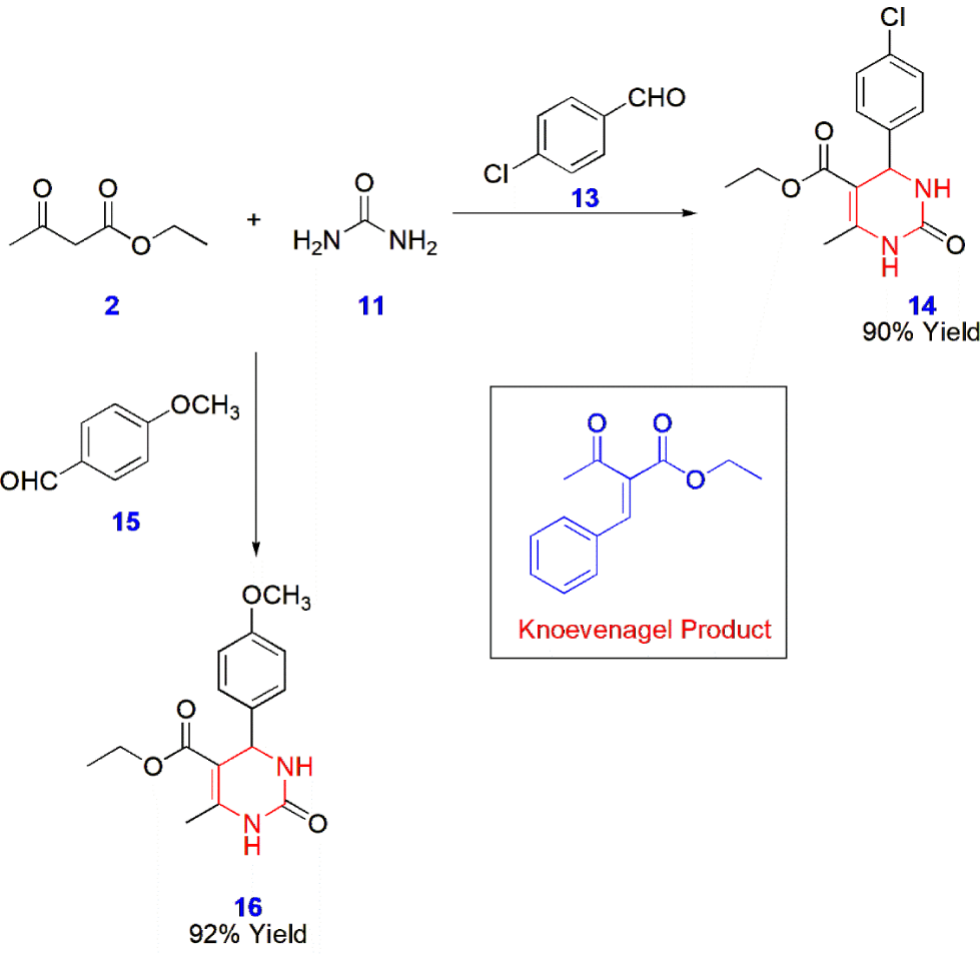
Synthesis of Dihydropyrimidines **14** and **16** via the Biginelli Reaction

**Scheme 4:**
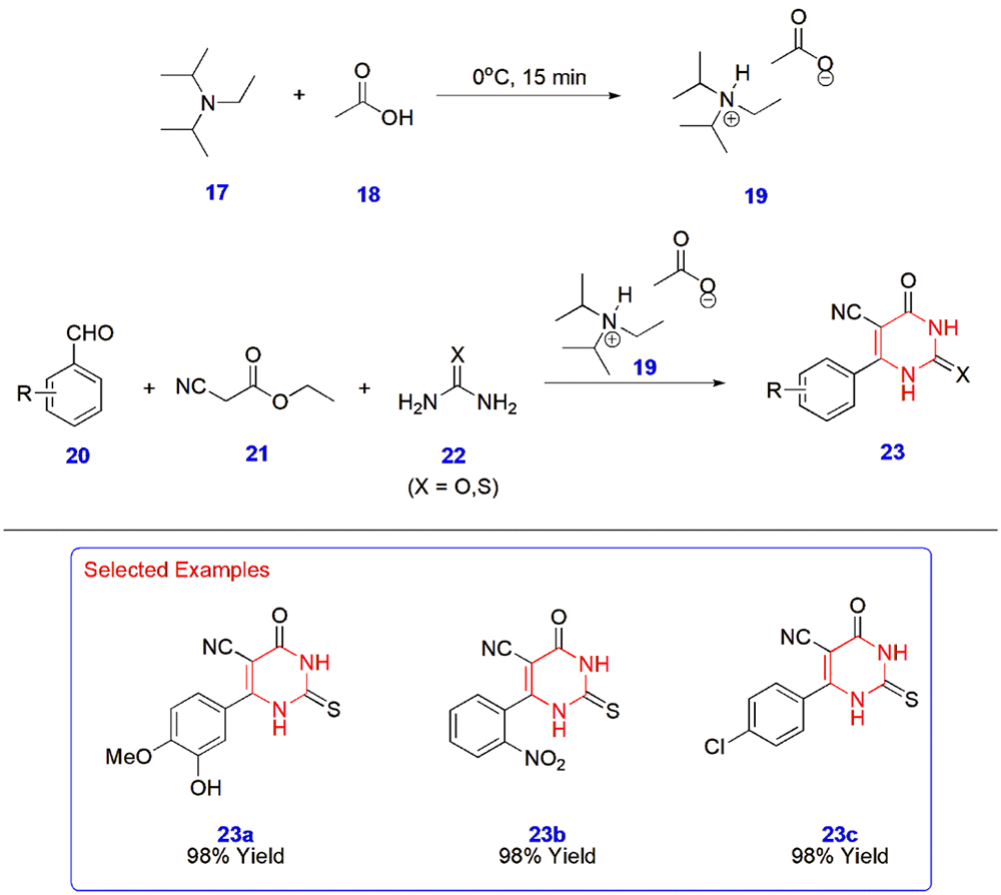
Biginelli Reaction for the Synthesis of 1,2,3,4-Tetrahydropyrimidines **23**

**Scheme 5:**
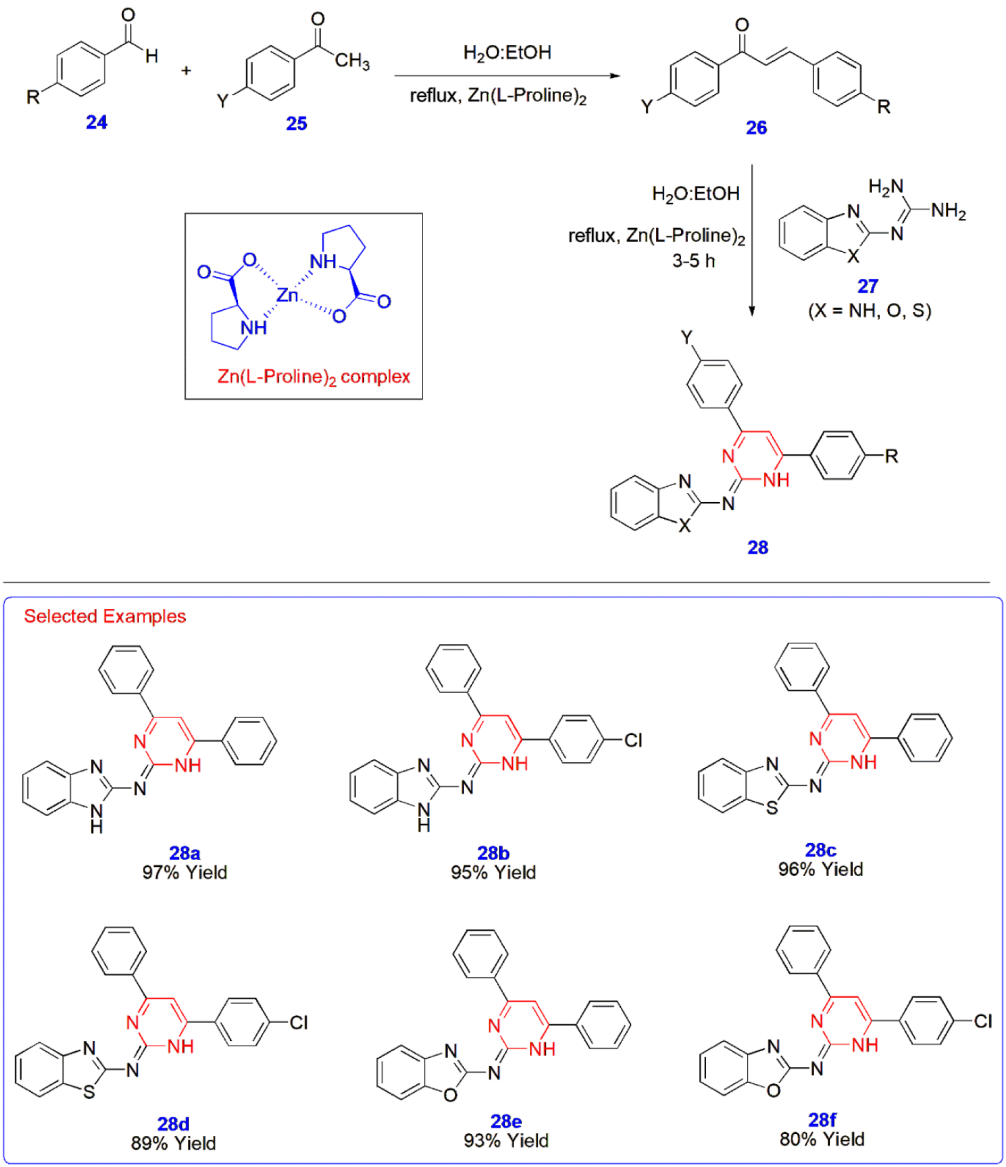
Zn Complex-Catalyzed Synthesis of Pyrimidines **28**

**Scheme 6:**
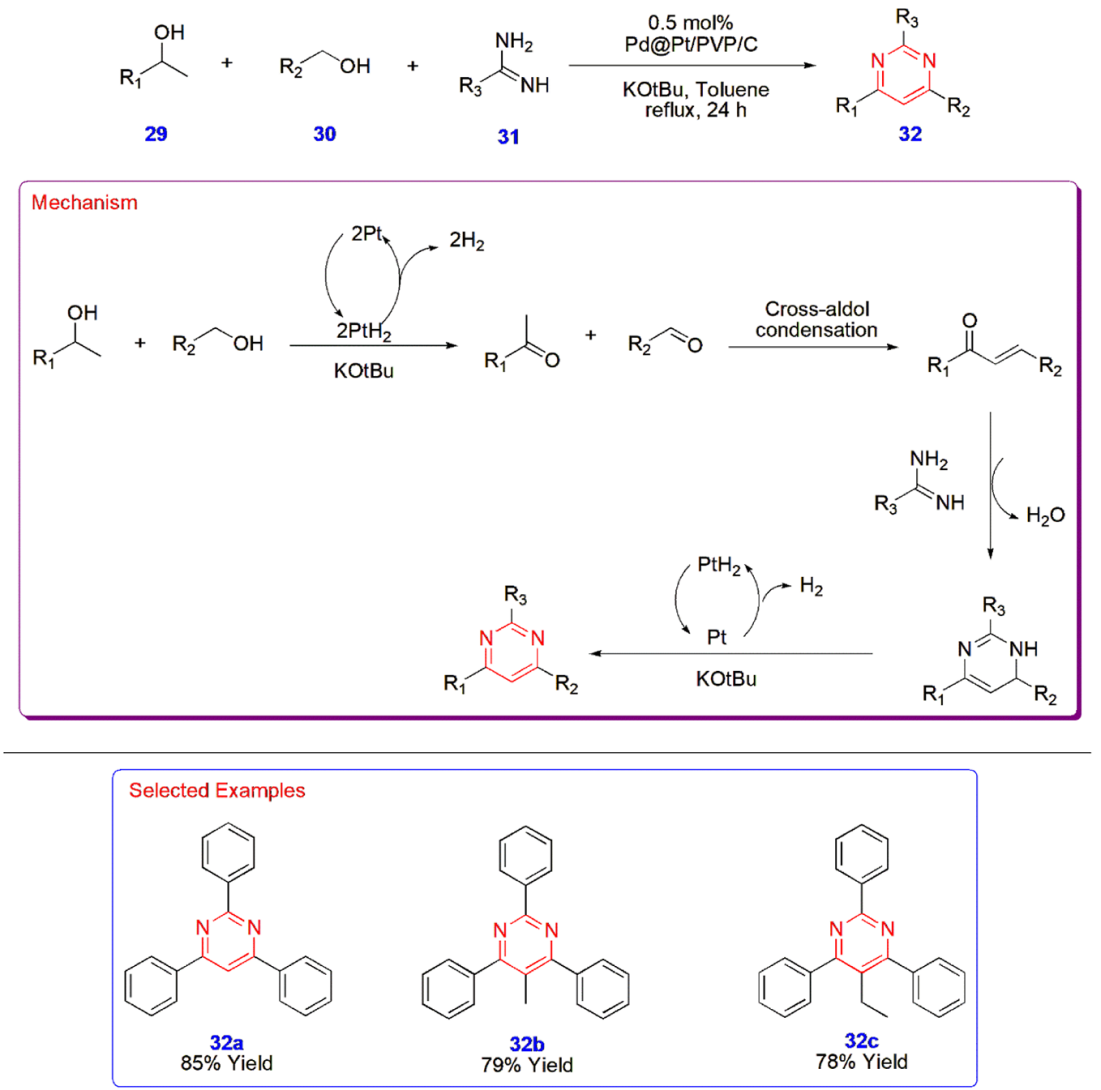
Palladium-Catalyzed ehydrogenativeDehydrogenation Synthesis of 2,4,6-Triphenyl Pyrimidines **32**

**Scheme 7:**
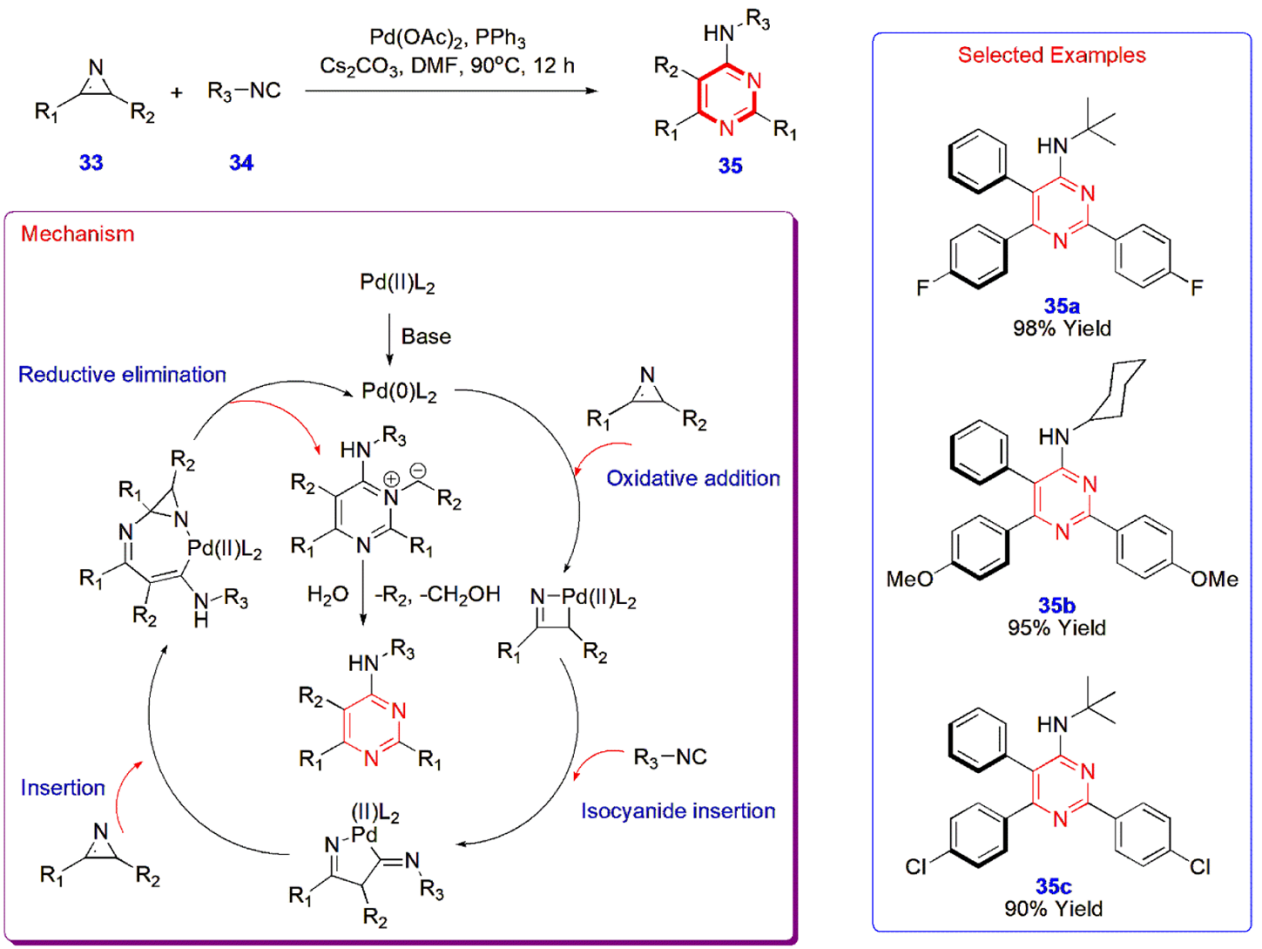
Palladium-Catalyzed Synthesis of Polysubstituted Pyrimidines **35**

**Scheme 8:**
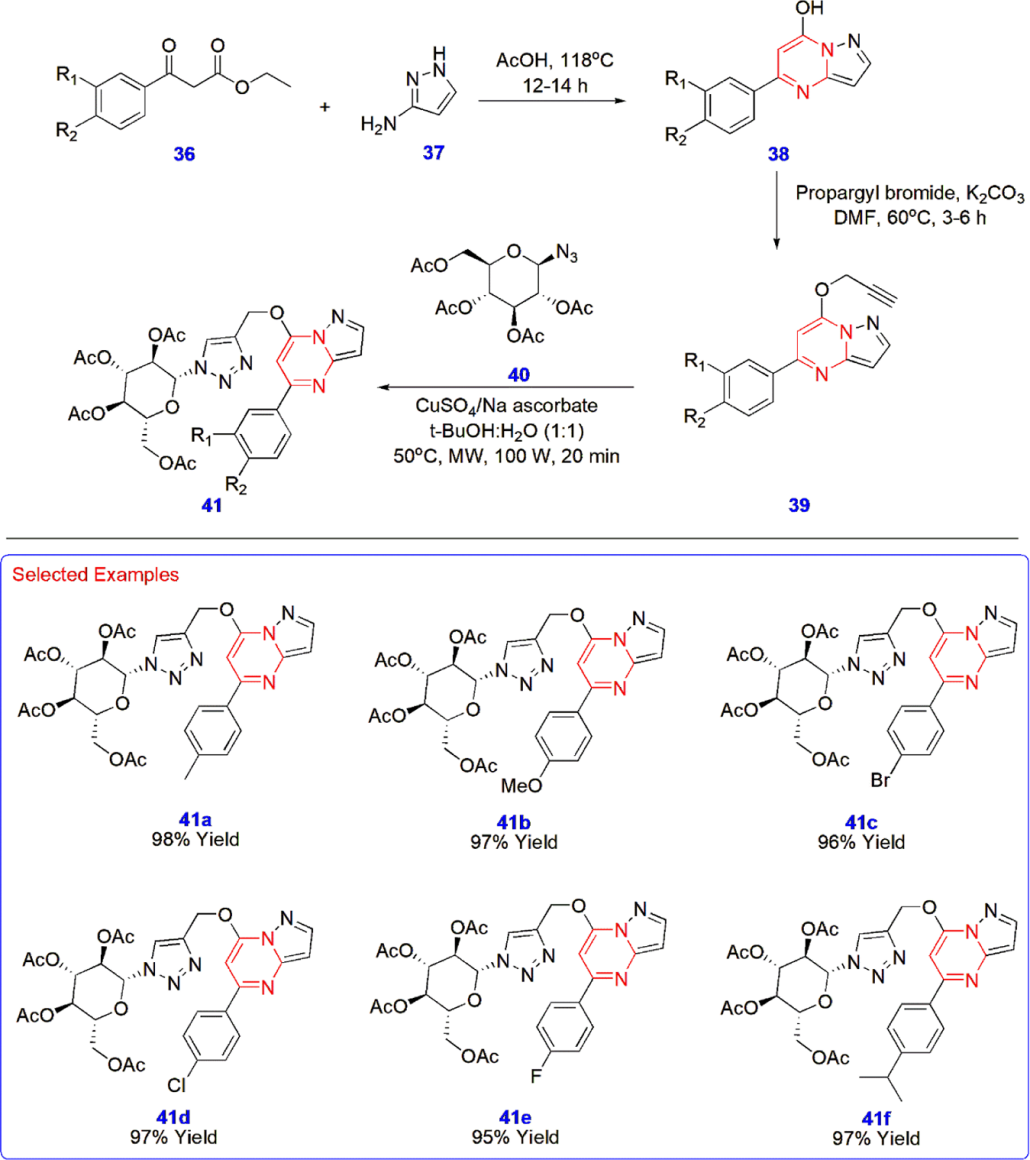
Synthesis of Pyrazolopyrimidine-Linked Triazole Glycohybrids **41**

**Scheme 9:**
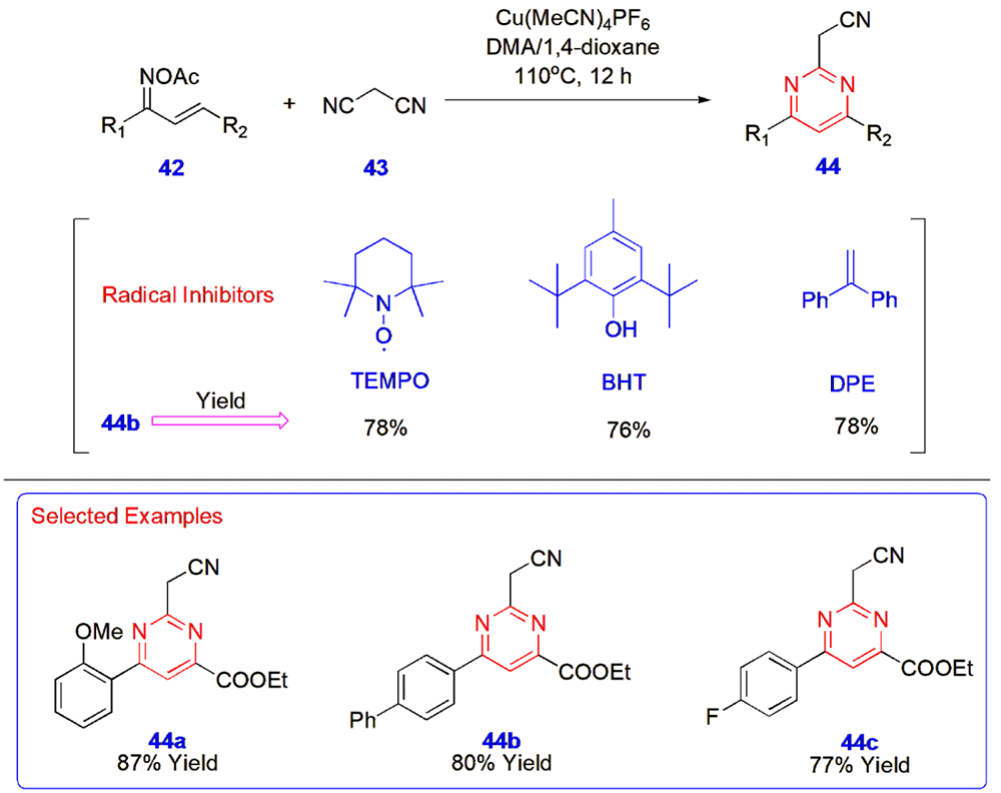
Copper-Catalyzed Synthesis of 2,4,6-Trisubstituted Pyrimidines 44 via [4+2] Annulation

**Scheme 10:**
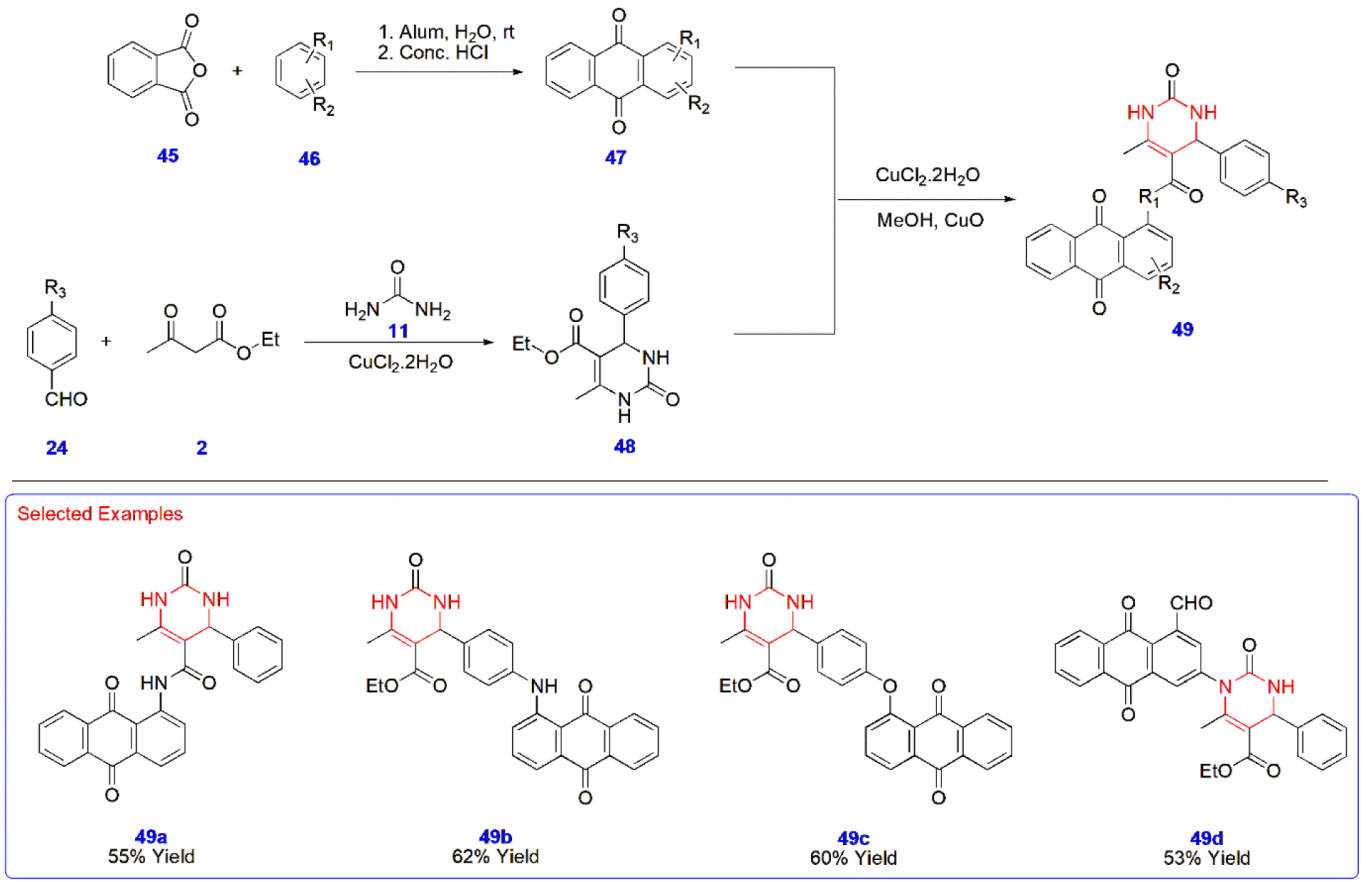
Copper-Catalyzed Synthesis of Anthraquinone-Based Pyrimidine Analogues **49**

**Scheme 11:**
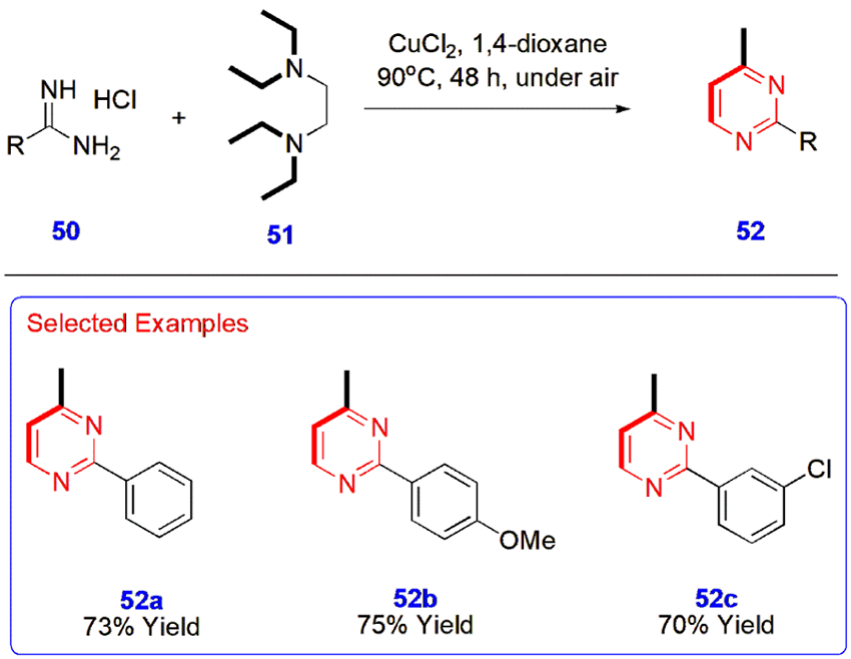
Copper-Catalyzed Difunctionalization of Tertiary Alkylamines for the Synthesis of Pyrimidines **52**

**Scheme 12:**
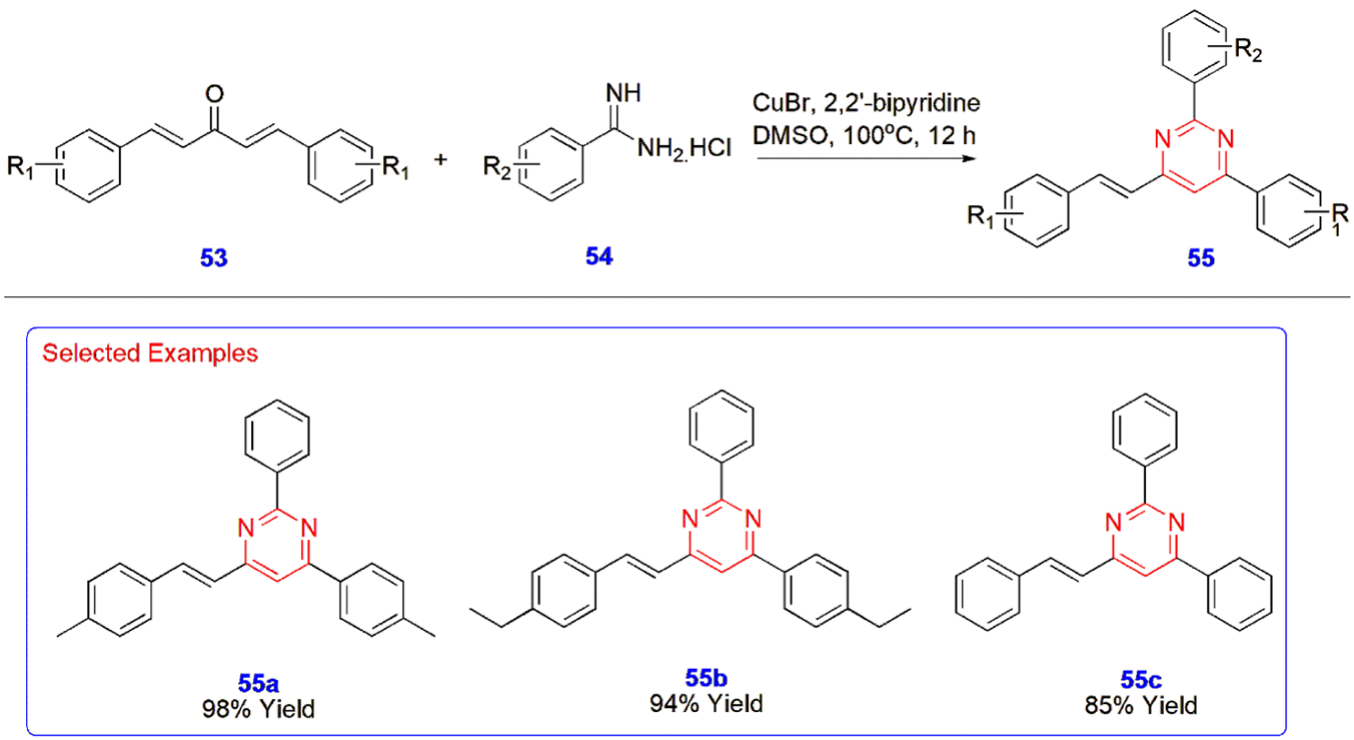
Synthesis of (*E*)-2,4-Diaryl-6-styrylpyrimidines **55**

**Scheme 13:**
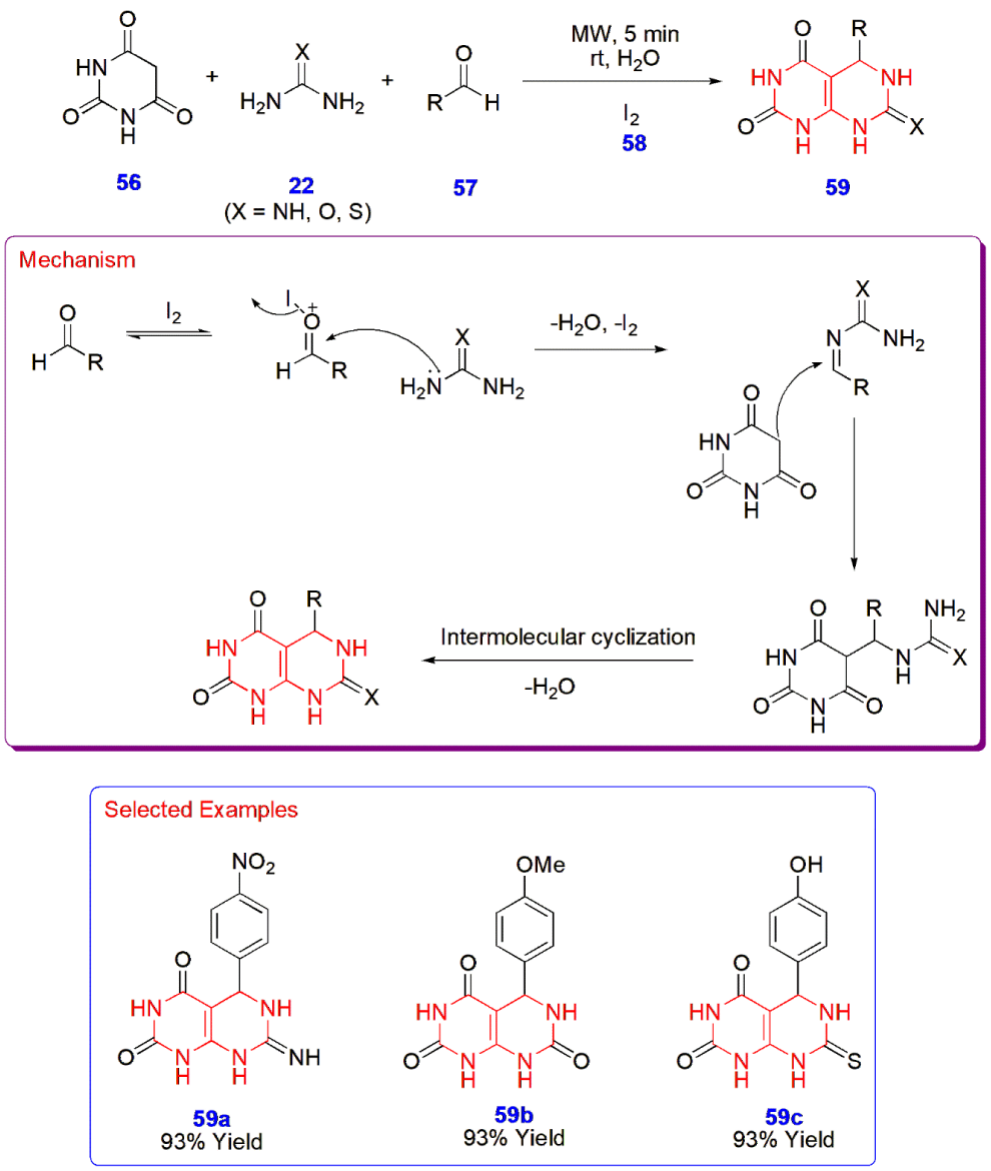
Microwave-Assisted Synthesis of Pyrimido­[4,5-*d*]­pyrimidines **59**

**Scheme 14:**
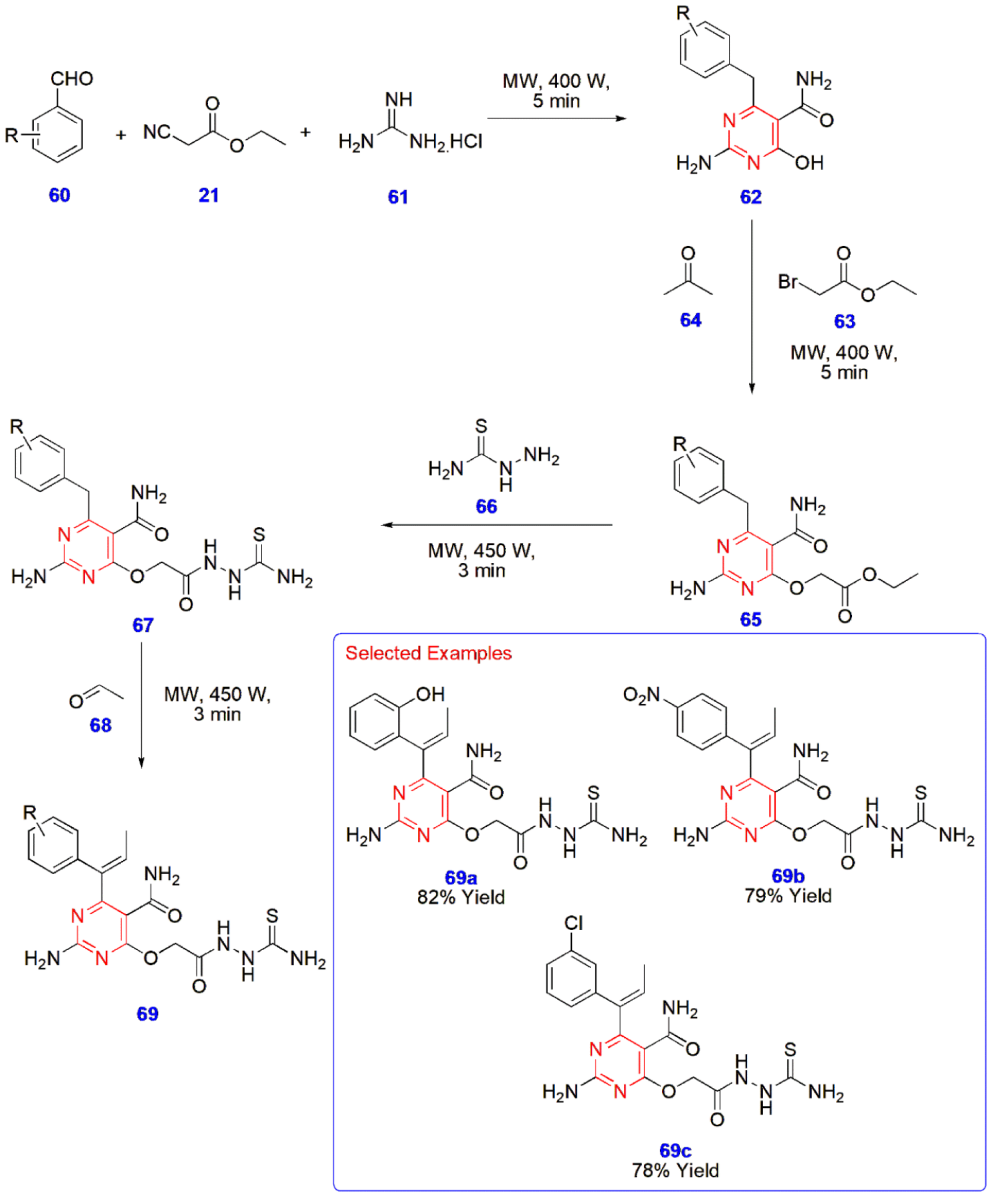
Microwave-Assisted Synthesis of Thiosemicarbazide Derivatives of Pyrimidine **69**

**Scheme 15:**
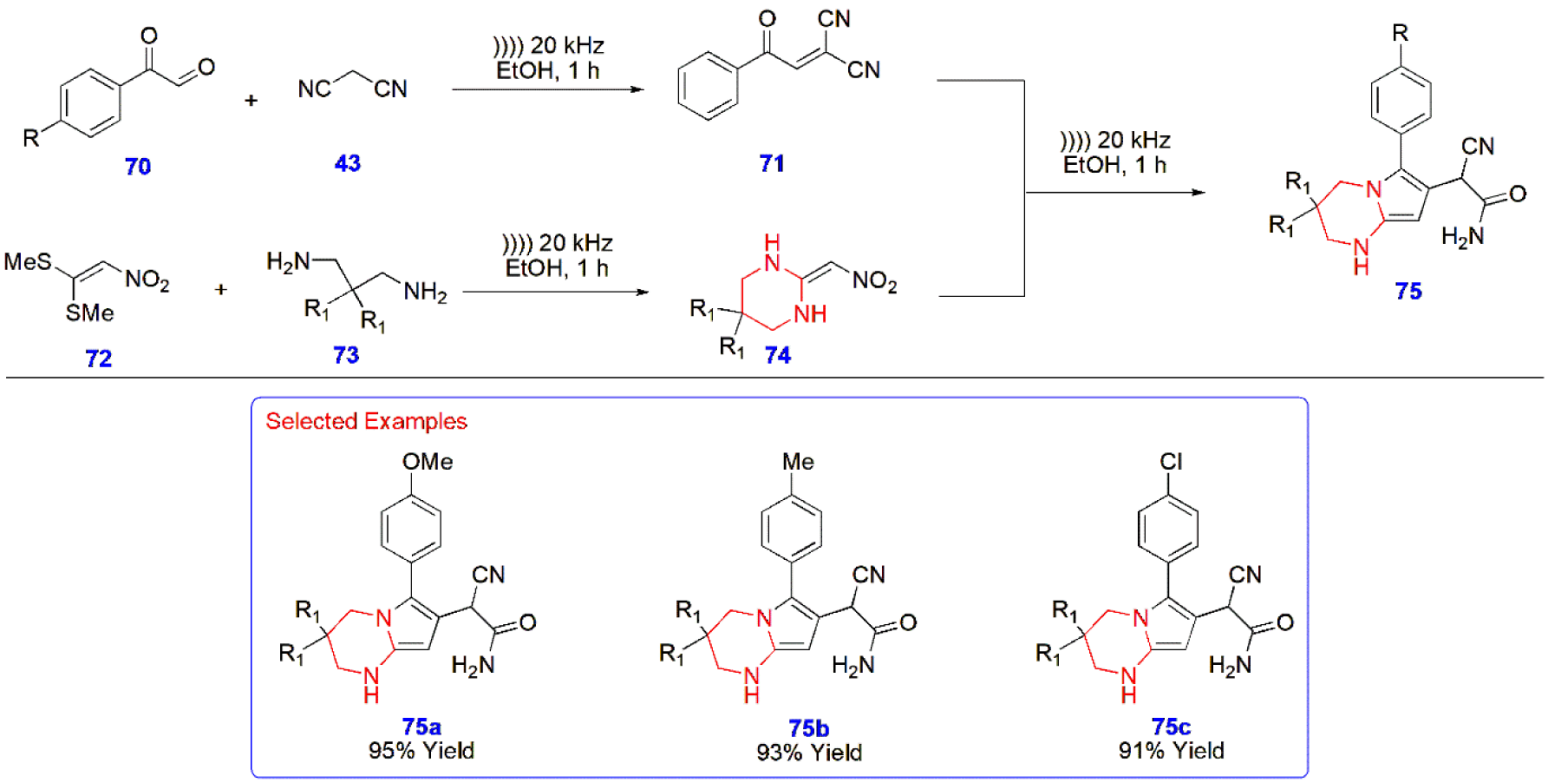
Ultrasound-Promoted Synthesis of Pyrrole-Fused Pyrimidine Analogues **75**

**Scheme 16:**
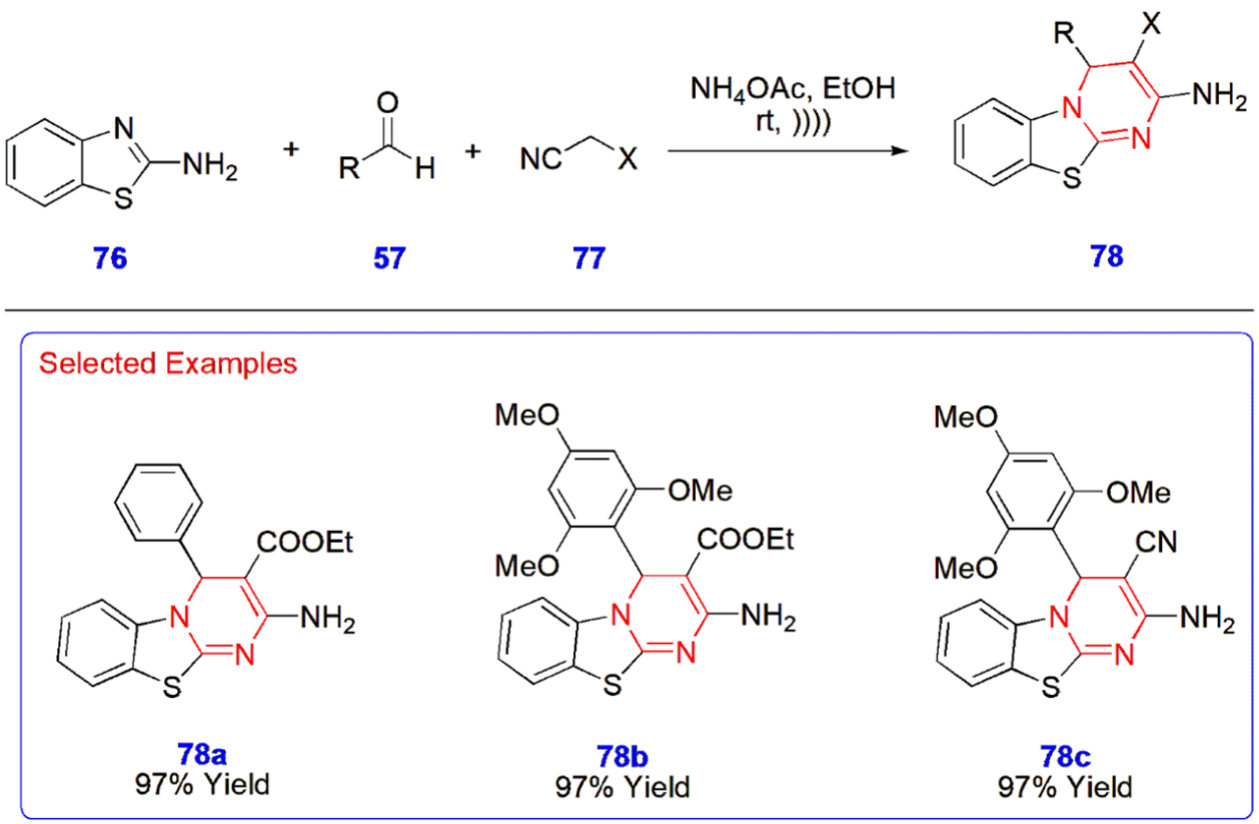
Ultrasound-Assisted Synthesis of Benzothiazole­[3,2-*a*]­pyrimidine Derivatives **78**

**Scheme 17:**
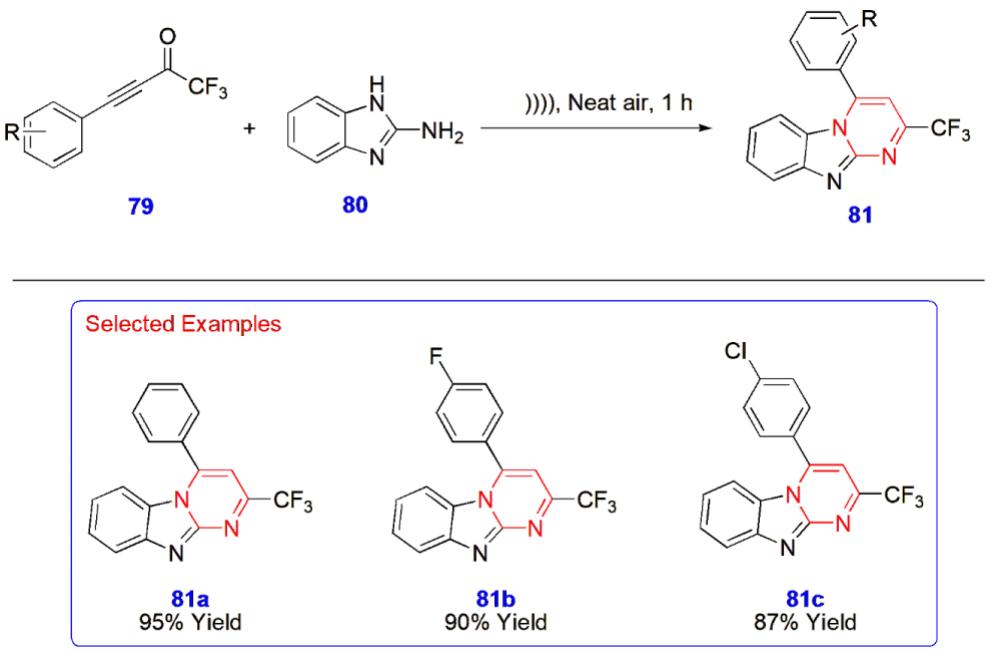
Ultrasound-Mediated Synthesis of Polyfluoro-pyrimido [1,2-*a*]­benzimidazole Analogues **81**

**Scheme 18:**
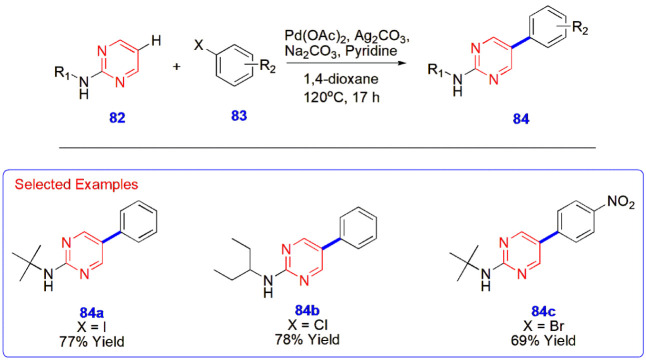
Palladium-Catalyzed C–H Functionalization of 2-Aminopyrimidines **82**

**Scheme 19:**
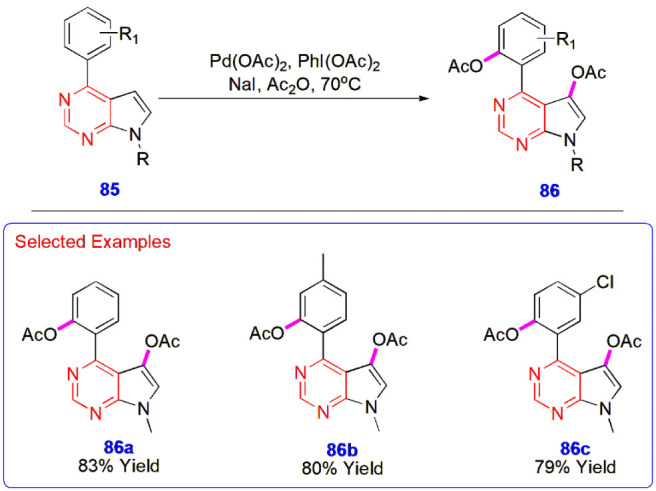
Palladium-Catalyzed, NaI-Promoted C–H Diacetoxylation of Pyrrolo­[2,3-*d*]­pyrimidine Derivatives **85**

**Scheme 20:**
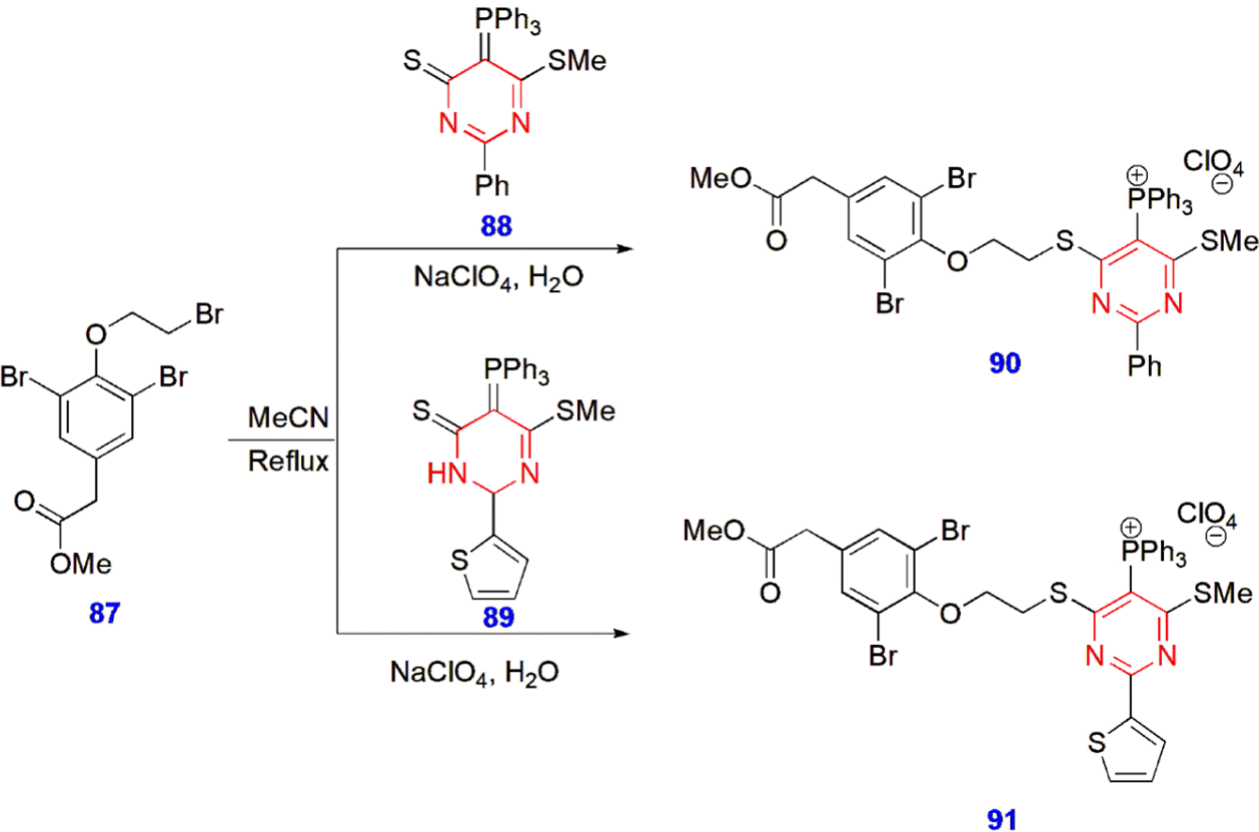
Synthesis of Triphenylphosphonium Functionalized Pyrimidines **90–91**

**Scheme 21:**
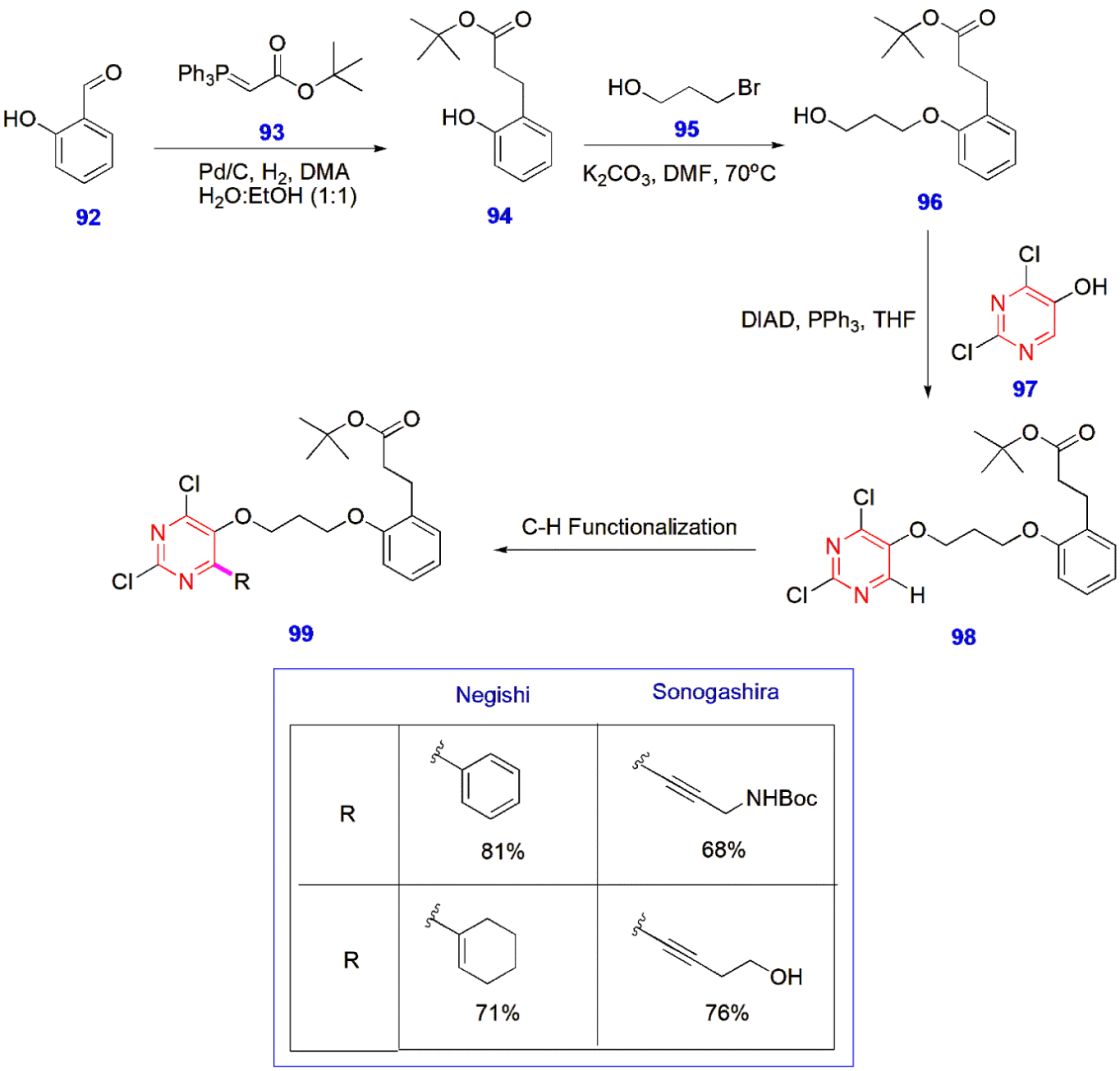
Synthesis and Functionalization of 2,4-Dichloropyrimidine Intermediate **97**

**Table 1:**
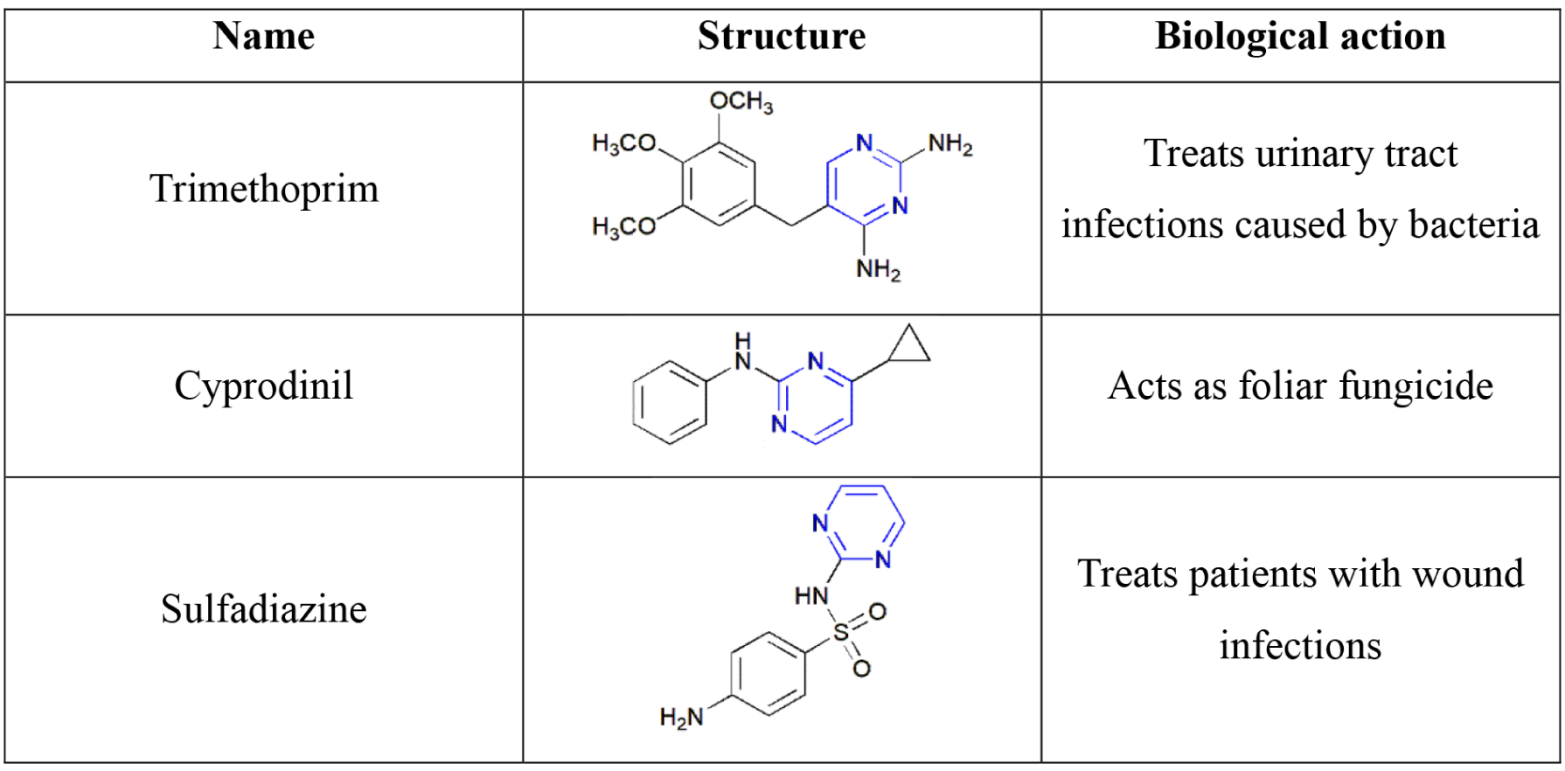
Pyrimidine-Containing Antimicrobia Drugs

**Scheme 22:**
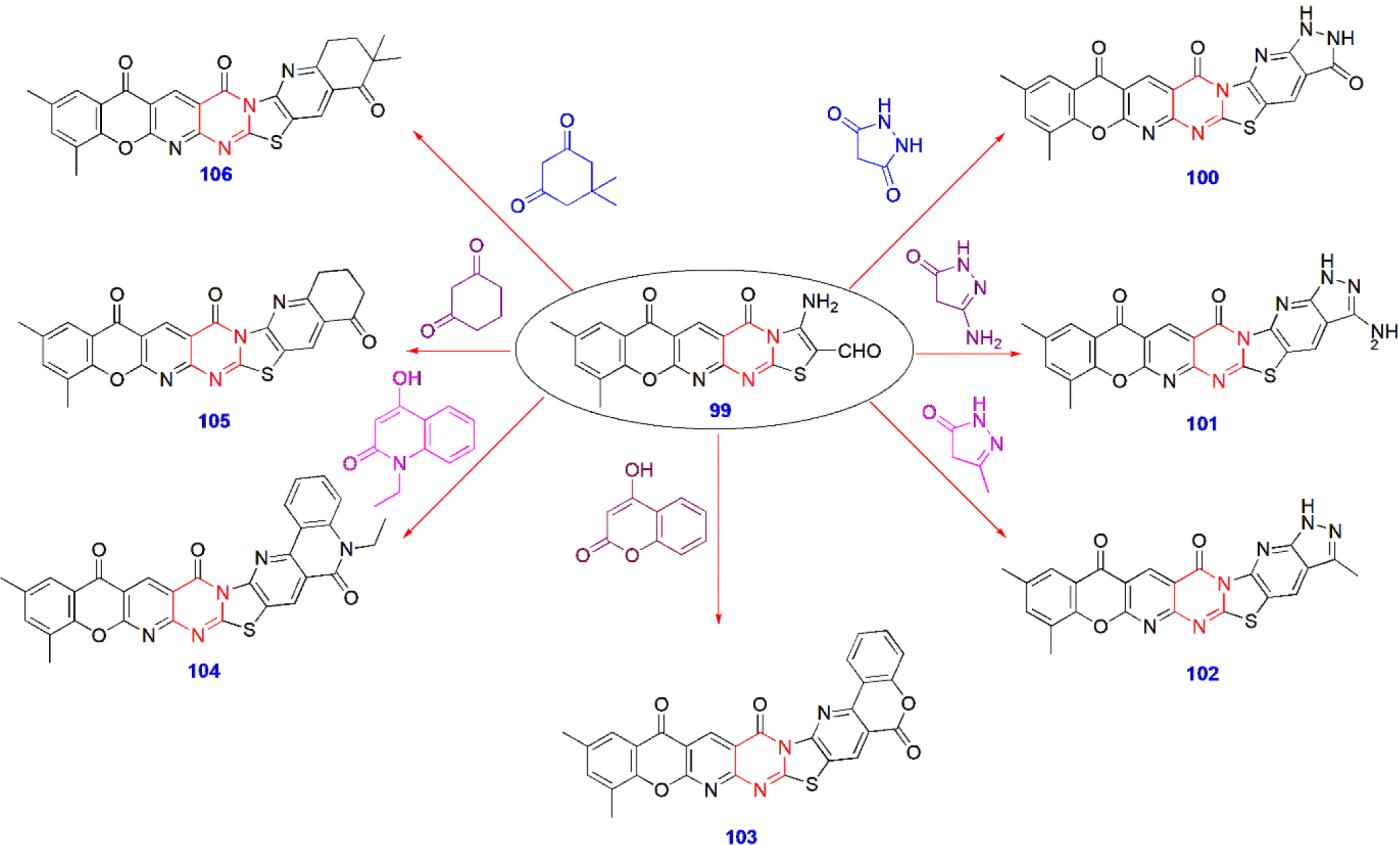
Synthesis of Antibacterial Compounds **100–106**

**Scheme 23:**
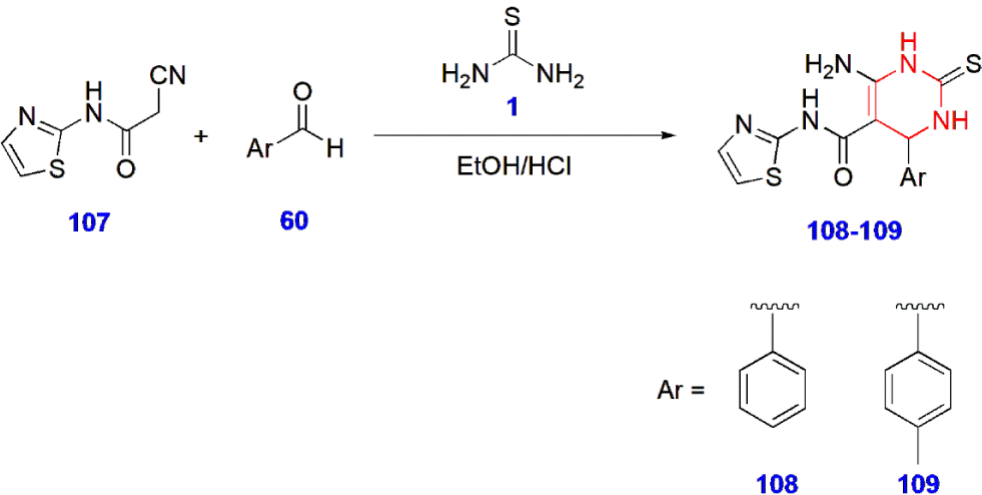
Synthesis of Thiazole-Linked Pyrimidine Derivatives **108–109**

**Scheme 24:**
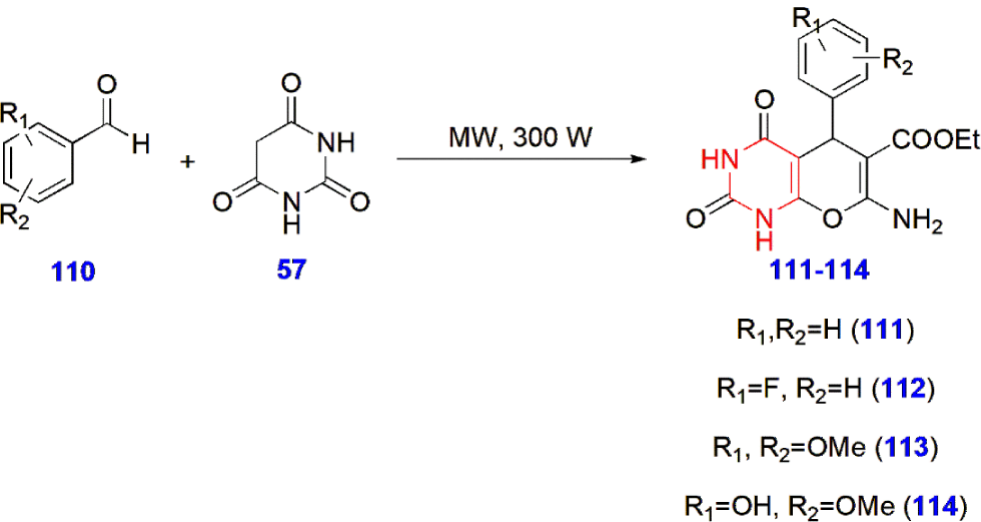
Microwave-Assisted Synthesis of Pyranopyrimidine Derivatives **111–114**

**Figure 3:**
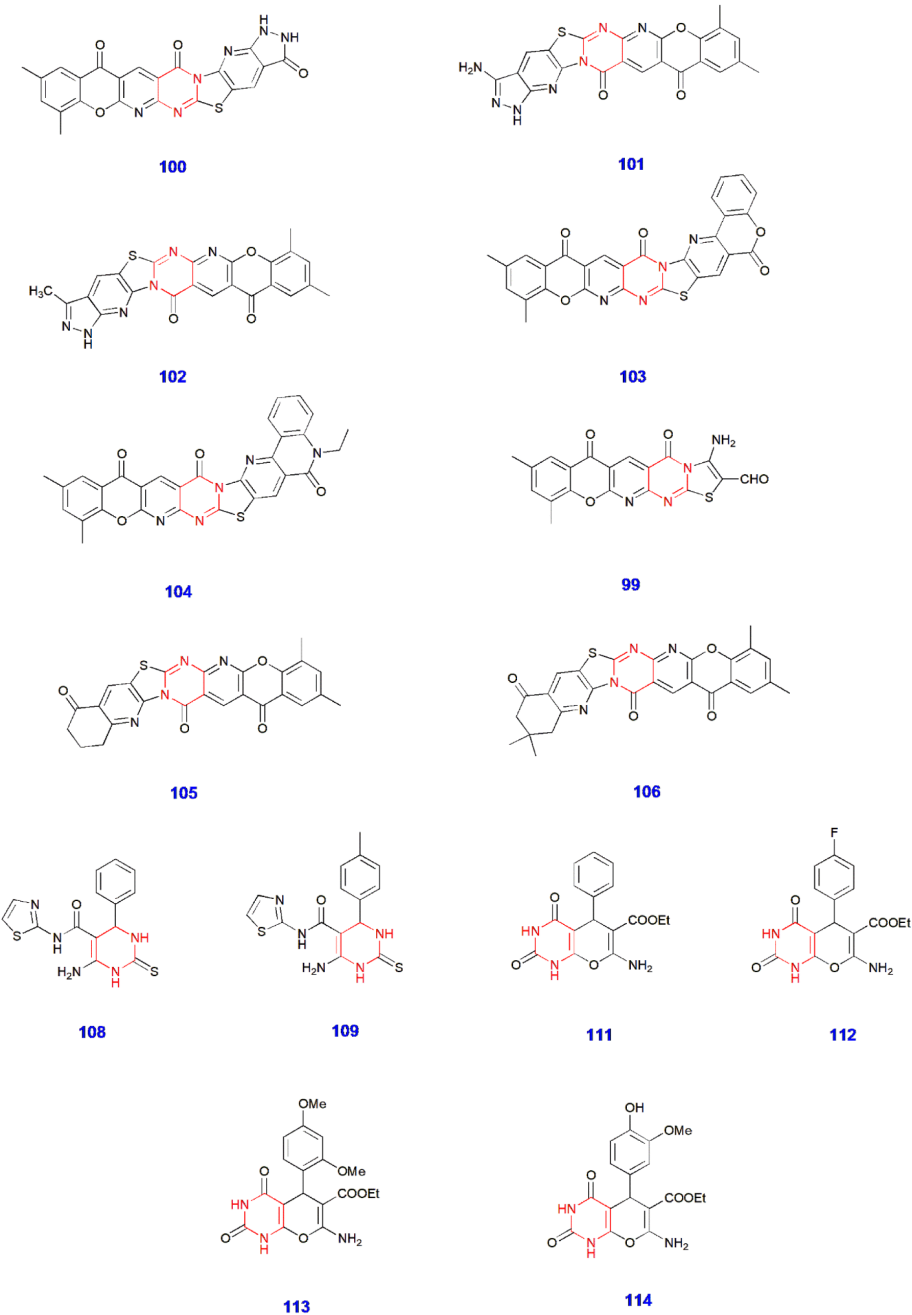
Pyrimidine-containing compounds showing antimicrobial activity.

**Scheme 25:**
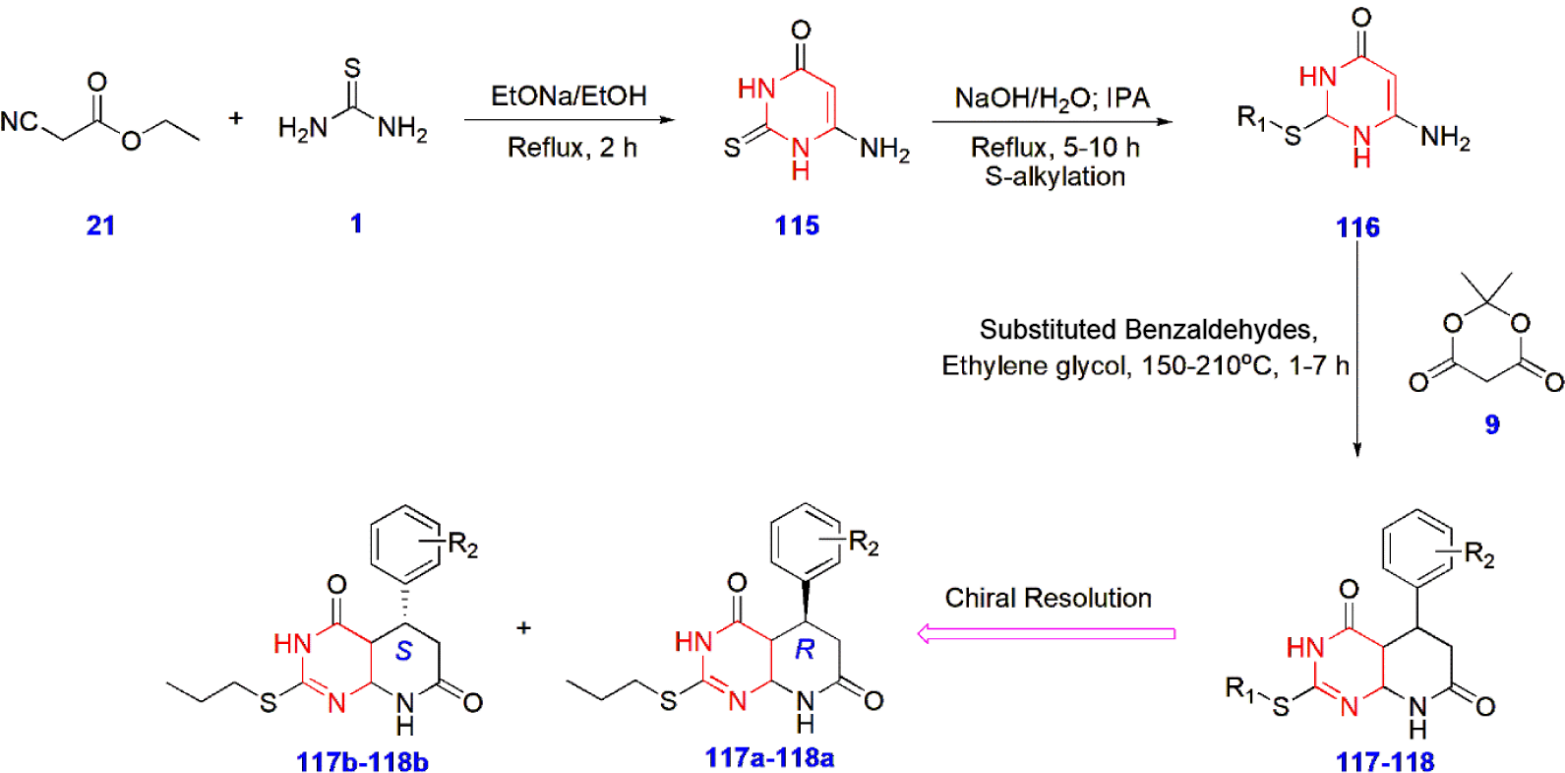
Synthesis of Pyrimidine Derivatives **117–118** with Chiral Resolution

**Scheme 26:**
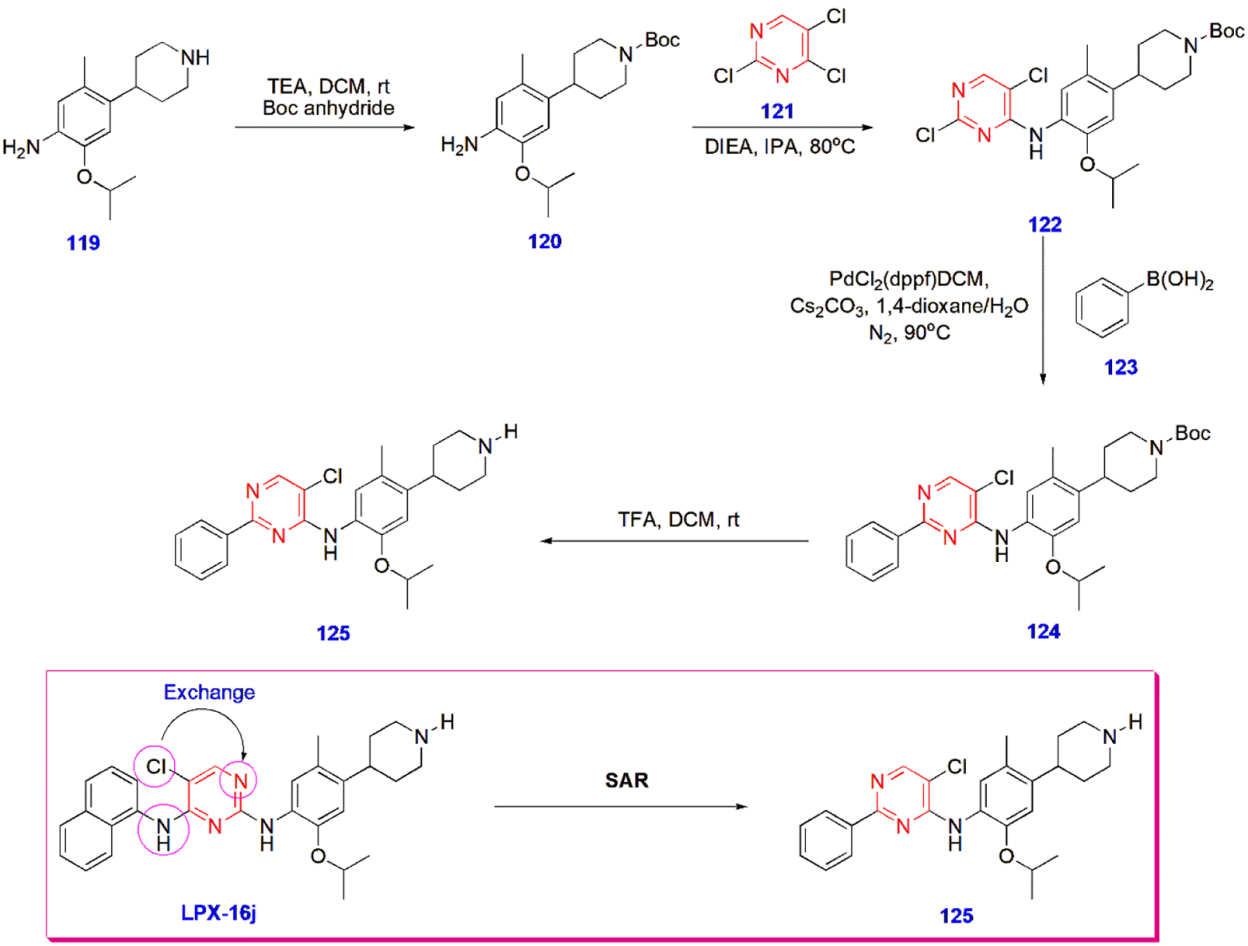
Synthesis of Novel Pyrimidine Derivative **125** for Antitubercular Activity

**Scheme 27:**
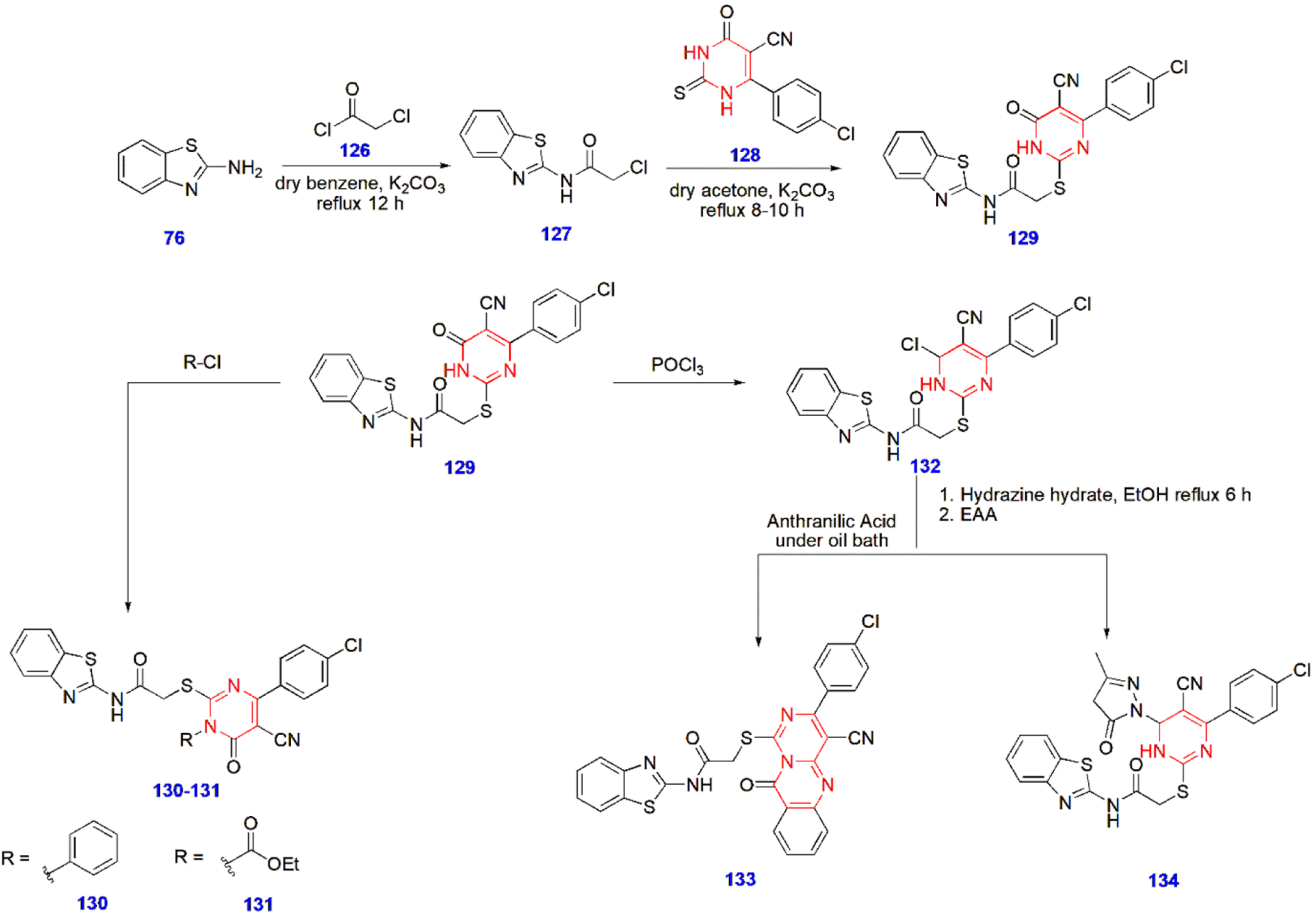
Synthesis of Pyrimidine-Linked Benzothiazole Analogues for Antitubercular Activity

**Figure 4:**
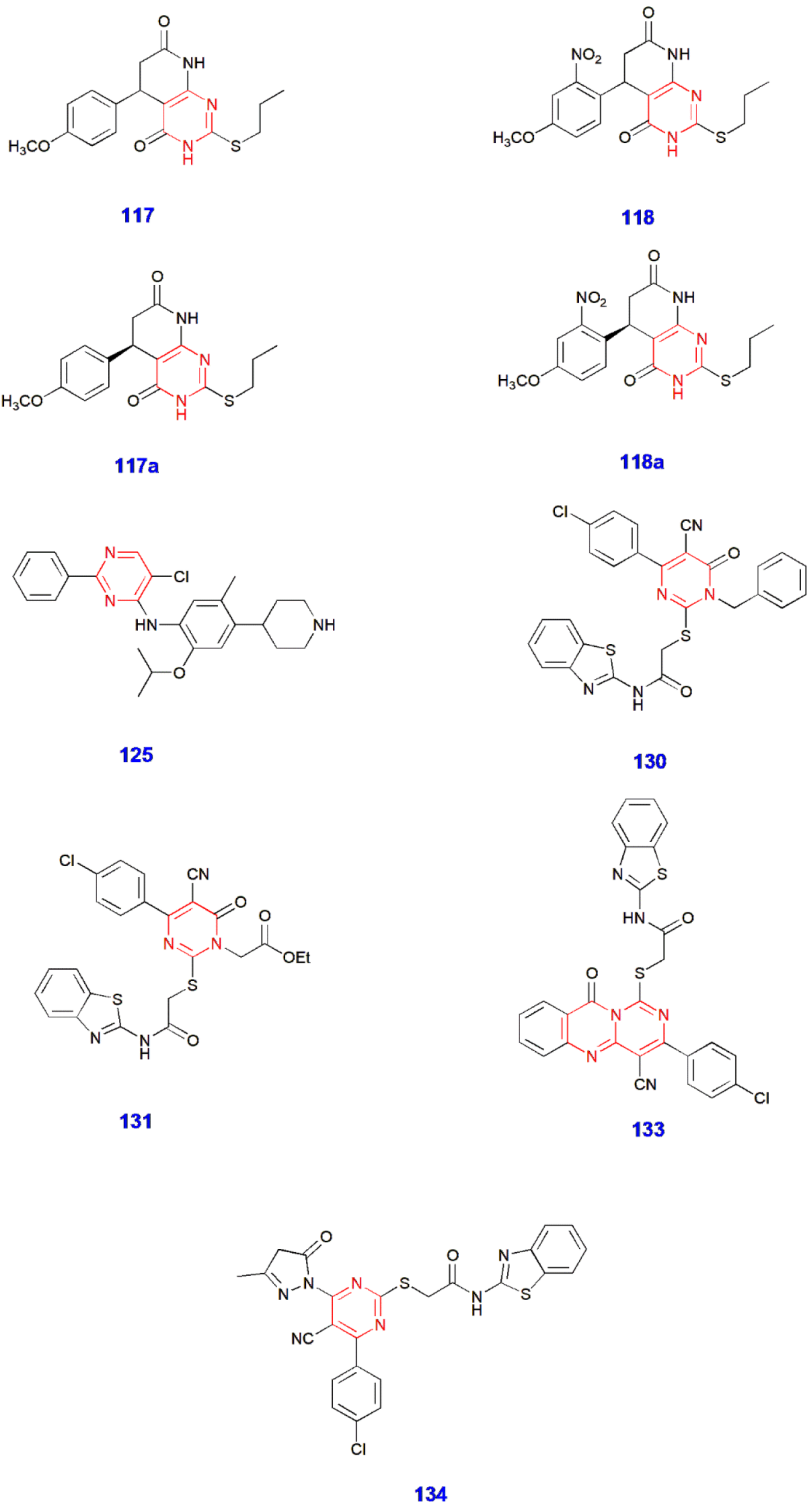
Pyrimidine-containing compounds showing antitubercular activity.

**Table 2:**
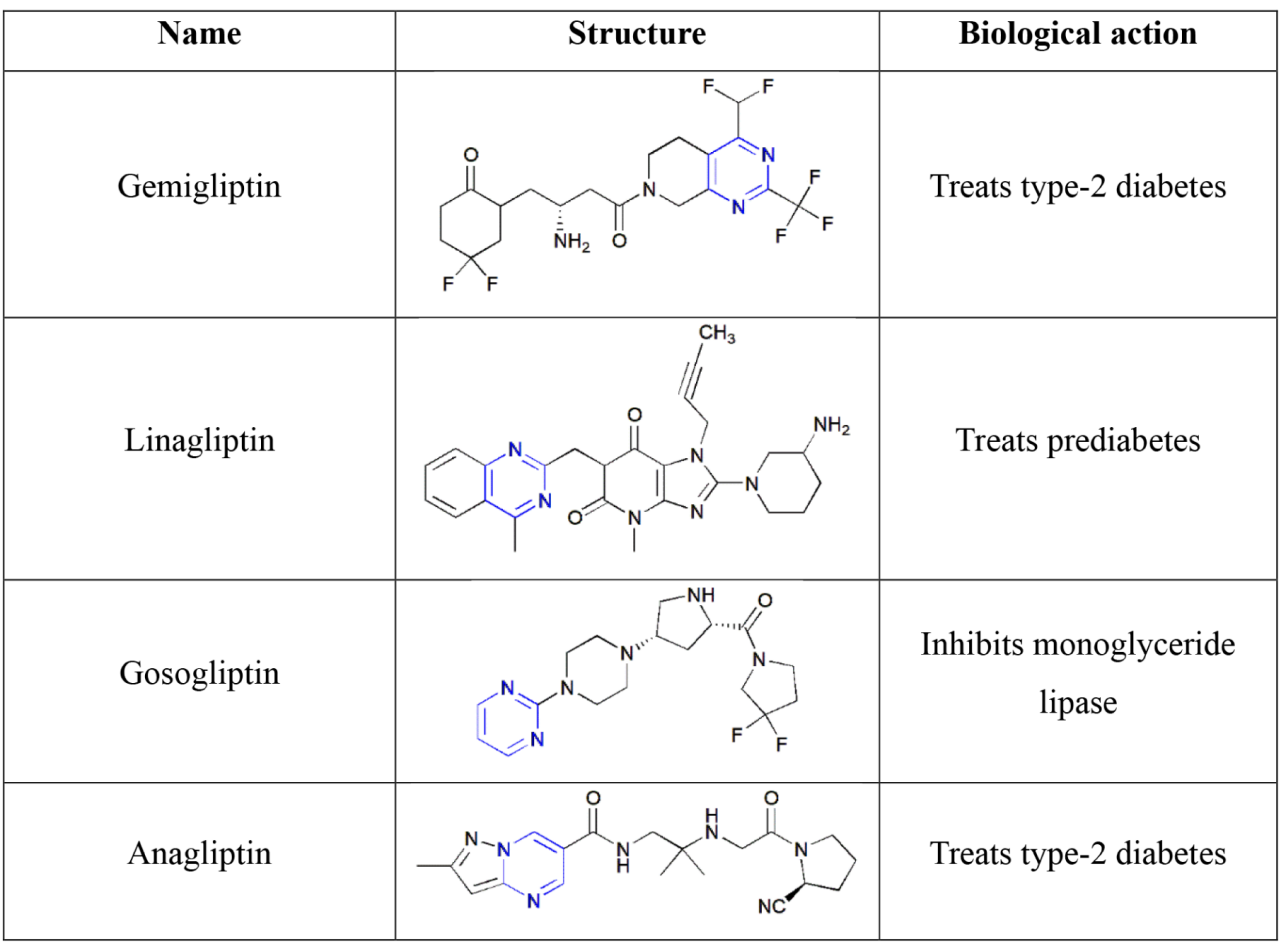
Pyrimidine-Containing Antidiabetic Drugs

**Scheme 28:**
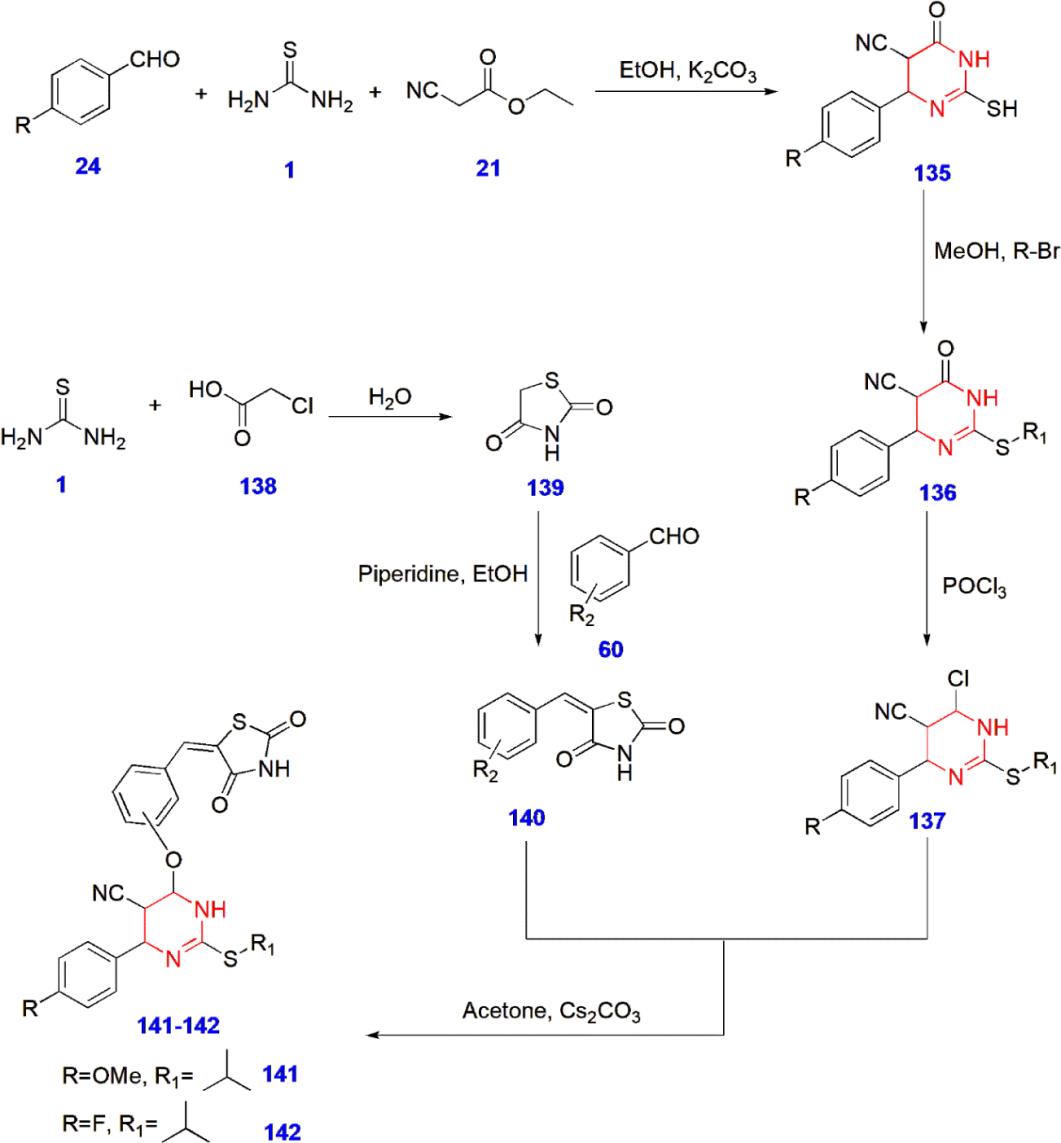
Synthesis of Thiazolidinedione-Linked Pyrimidine Derivatives **141–142** for Antidiabetic Activity

**Scheme 29:**
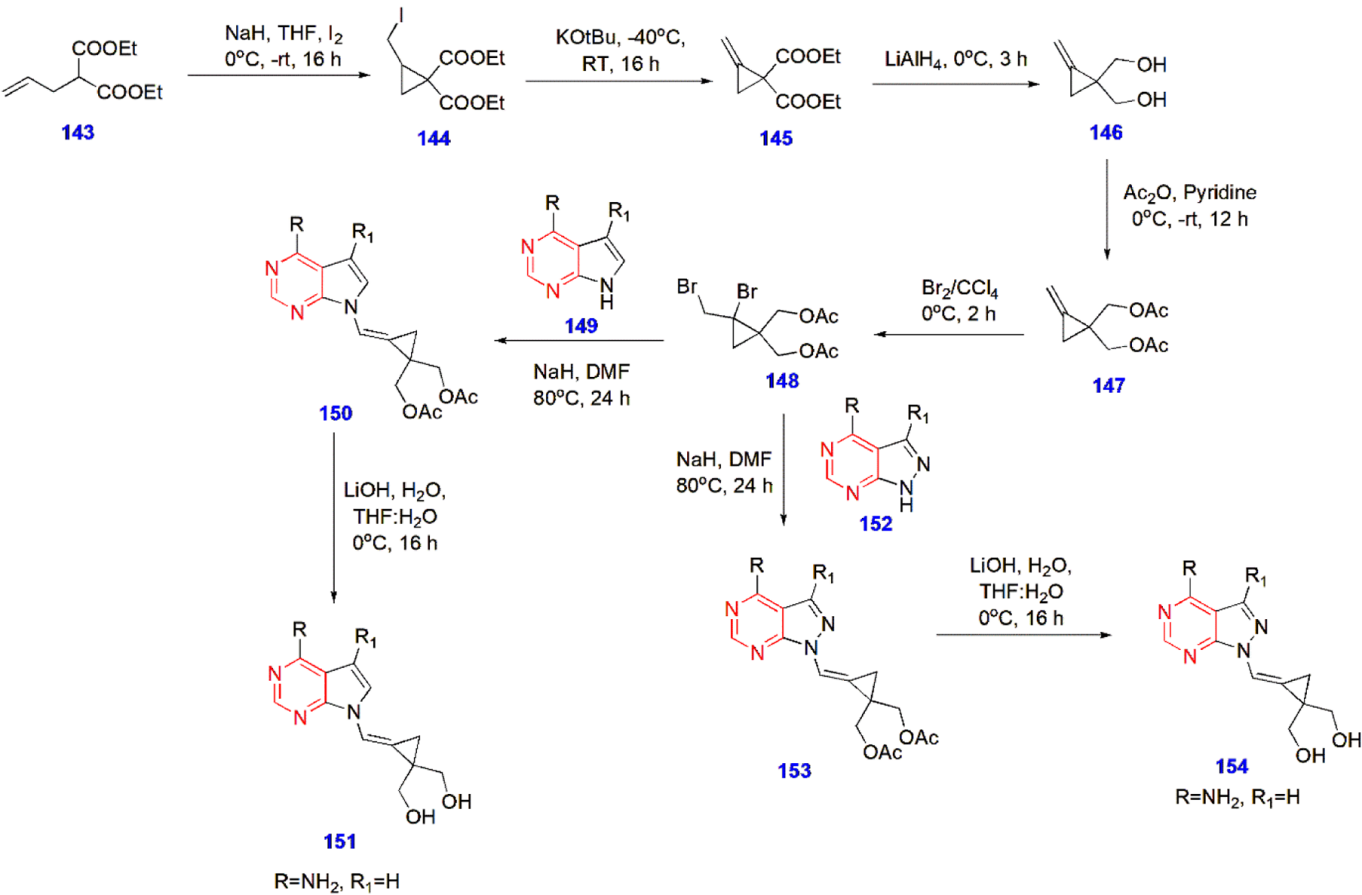
Synthesis of Pyrimidine-Tethered Carbocyclic Nucleoside Analogues **151** and **154** for Antidiabetic Activity

**Scheme 30:**
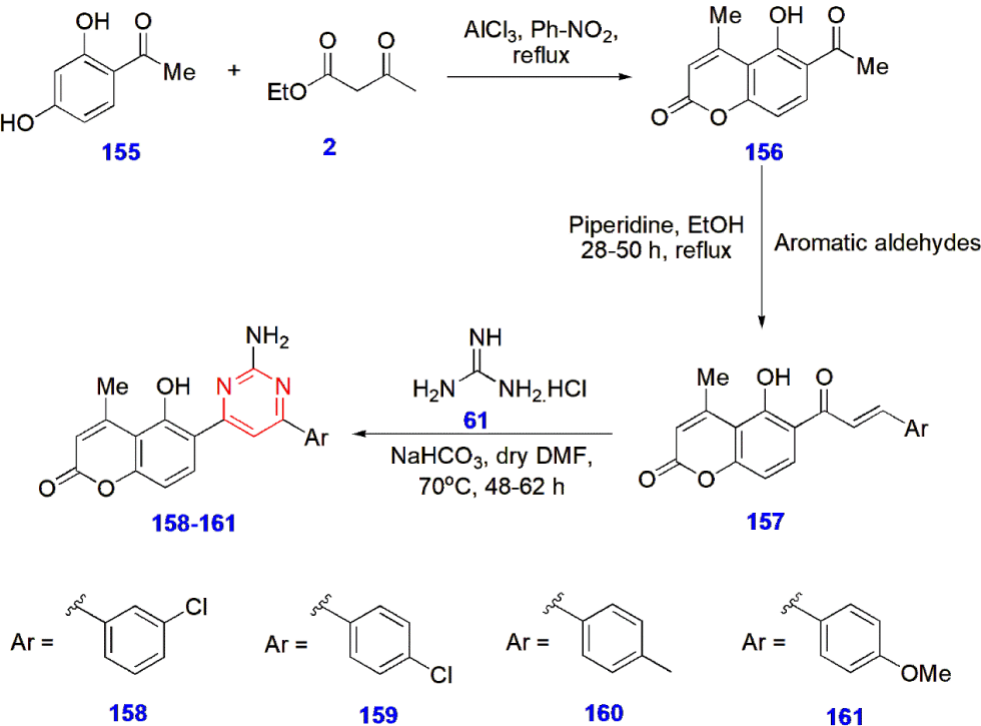
Synthesis of Pyrimidine-Coumarin Hybrids **158–161** for Antidiabetic Activity

**Figure 5:**
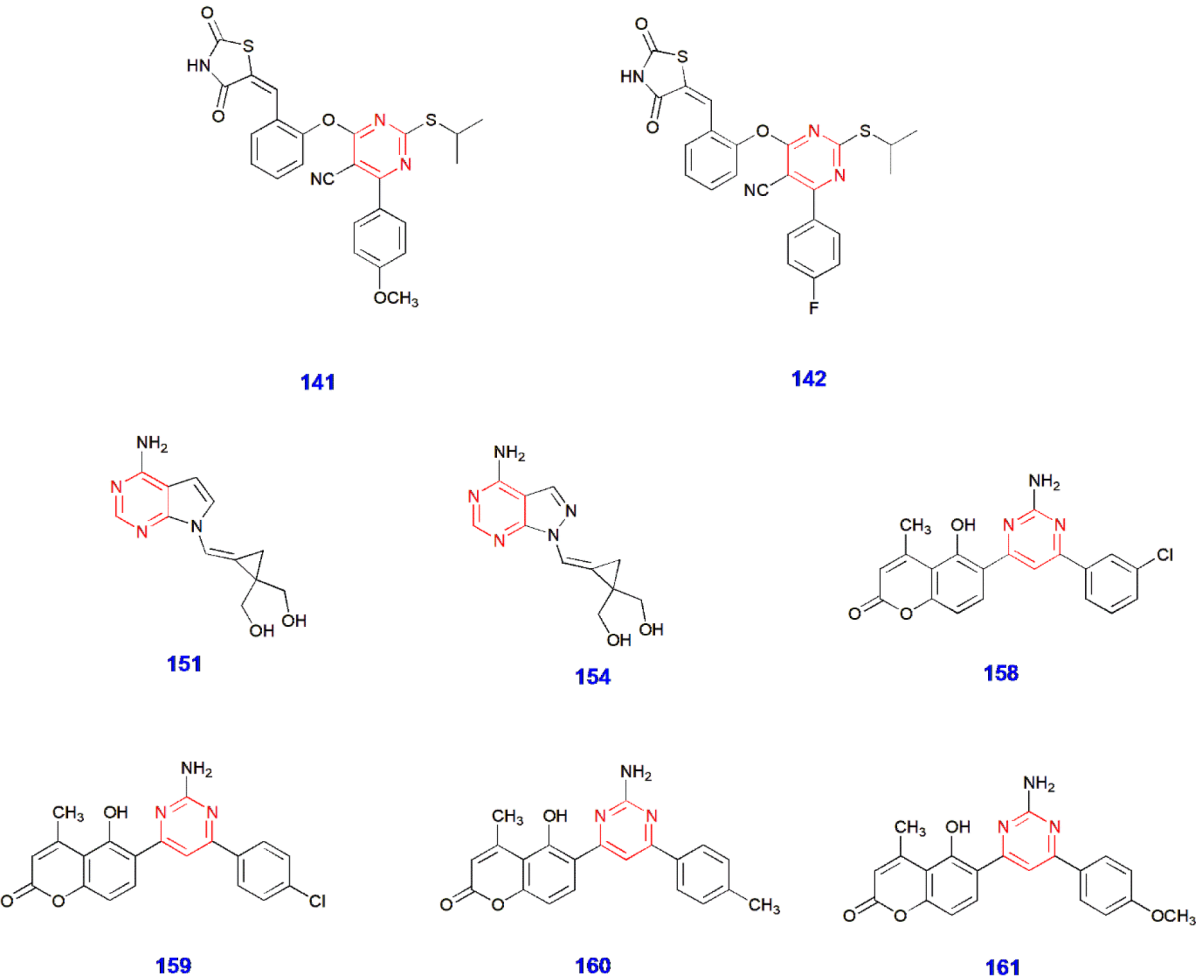
Pyrimidine-containing analogues showing antidiabetic activity.

**Table 3:** Overall Comparison of Biologically Active Compounds

Sl. No	Biological Activity	Compound No.	Biological Action	Reference
1.	Antimicrobial	99	Inhibition of *Aspergillus fumigatus* (antifungal agents)	^69^
2.		100		
3.		101	Inhibition of *S. aureus*, *B. subtilis*, *S. typhimurium*, *E. coli* (antibacterial agents)	
4.		102		
5.		103		
6.		104	Inhibition of *S. aureus*, *B. subtilis* (antibacterial agents)	
7.		105		
8.		106	Inhibition of *Aspergillus fumigatus* (antifungal agent)	
9.		108	Inhibition of *S. aureus*, *S. faecalis*, *E. coli*, *K. pneumoniae*, *S. cerevisiae* (antibacterial and antifungal agents)	^70^
10.		109		
11.		111	Inhibition of *Bacillus*, *E. coli*, *Pseudomonas*, *Candida*, and *Aspergillus* (antibacterial and antifungal agents)	^71^
12.		112		
13.		113		
14.		114		
15.	Antitubercular	117	Inhibition of *M. tuberculosis* H37Rv and *M. marinum* (antitubercular agents)	^72^
16.		117a		
17.		118		
18.		118a		
19.		125	Inhibition of *M. tuberculosis* H37Ra and H37Rv (antitubercular agent)	^73^
20.		130	Inhibition of *M. tuberculosis* (ATCC 25177) (antitubercular agents)	^74^
21.		131		
22.		133		
23.		134		
24.	Antidiabetic	141	Improvement of hyperglycemic conditions in diabetic patients (antidiabetic agents)	^81^
25.		142		
26.		151	Effective against α-glucosidase (antidiabetic agents)	^82^
27.		154		
28.		158	Effective against α-amylase (antidiabetic agents)	^83^
29.		159		
30.		160	Effective against α-glucosidase (antidiabetic agents)	
31.		161		

Several metal-catalyzed reactions are instrumental in the synthesis of pyrimidines. The selection, improvisation, and incorporation of these compounds are important for obtaining target molecules in significant yields; hence, recent advances and research on pyrimidine chemistry are needed.^22^ Likewise, microwave- and ultrasound-assisted organic synthesis have been widely used in recent years.^23–25^ Their utility has been remarkable in the production of several distinguished heterocyclic scaffolds, and their scale-up reactions and process chemistry remain challenging.

In recent years, extensive research has been carried out on the direct functionalization of heterocyclic compounds, such as C–H and N–H. These functionalization processes involve different molecules, and their productivity is high.^26–29^ The biology of pyrimidines is being studied in all sectors of education and research, as it is related to genetic approaches and human life. Various researchers working on pyrimidine molecules have pushed themselves to produce compounds with therapeutic potential, such as antiviral,^30,31^ anticancer,^32,33^ anti-inflammatory,^34,35^ and antioxidant agents.^36,37^ Apart from these, pyrimidine-containing heterocyclic scaffolds have demonstrated their utility as anti-HIV, antihypertension, and antimalarial agents.^38^ Nowadays, pyrimidines are used for the detection of SARS-CoV-2 via triplex formation of modified bis-pyrimidine core structures.^39^ Multifaceted studies are being carried out beyond the biology and synthetic fields of pyrimidine, taking them to newer dimensions.^40^ In our previous work, we discussed the latest developments in the biology of pyrimidines in the above-mentioned areas, along with a few synthetic methods (Figure 2).^41^

Since the versatility of pyrimidine has grown to greater heights, the aim or target of this review is to focus on the latest progress in the synthetic chemistry and biological importance of pyrimidine-containing heterocyclic hybrids. With this intent, we present the recent developments in the synthetic chemistry of pyrimidines involving Biginelli reactions, metal-catalyzed reactions, microwave- and ultrasound-promoted syntheses, and different direct functionalization reactions for the development of various analogues of therapeutic importance. In addition to their synthetic protocols, the latest results concerning antimicrobial, antitubercular, and antidiabetic activities have been included, which will direct the scope of this article to drug design and development. The detailed discussions and overviews help synthetic and medicinal chemists to dive deep into pyrimidine-containing heterocyclic chemistry. Therefore, this article benefits researchers working in both academic and industrial areas.

## 2 Synthetic Developments of Pyrimidines

### 2.1 Biginelli Reactions

Biginelli reactions have been instrumental in producing several interesting dihydropyrimidines via an acid-catalyzed three-component reaction between an aldehyde, β-ketoester, and various urea derivatives. This has been a classic approach for producing biologically active pyrimidines as well. Here, we discuss the latest progress in pyrimidine-related synthetic chemistry via Biginelli reactions under different reaction conditions.

Zohny et al. used the Biginelli reaction for the synthesis of pyrimidine derivatives **8,** which are suspected to be promising calcium channel blockers for lowering human blood pressure, thereby acting as antihypertensive agents. By incorporating aromatic aldehydes, several optimizations were carried out, among which 1,3-diphenyl-1*H*-pyrazole-4-carbaldehyde **3** results in active calcium channel blockers with pyrimidine core structures. Moreover, their synthesis started with the reactions of thiourea **1**, ethyl acetoacetate **2**, and the aforementioned aryl aldehyde **3** to yield the corresponding pyrimidine ester **4**. This ester was then hydrolyzed via alcoholic NaOH to acid **5** and transformed into the chloro derivative **6** via thionyl chloride. As the final step, the resulting chloro derivatives were condensed with three different aromatic amines **7** namely, aniline, *p*-toluidine and *p*-nitroaniline to yield the desired final compounds **8** (Scheme 1).^42^

Venkatesh et al. constructed [1,3]­dioxino­[4,5-*d*]­pyrimidine derivative **12** via the Biginelli reaction involving Meldrum’s acid **9**, indole-3-carbaldehyde **10,** and urea **11** in ethanol. The most interesting aspect of this reaction was its completion within 20 min under reflux, facilitated by ceric ammonium nitrate (CAN). The mechanism involves the formation of the Schiff base intermediate and its reaction with Meldrum’s acid **9** as the reactive methylene compound, triggering the formation of pyrimidines with the loss of a water molecule. The product held its own significance as an antitubercular agent tested against the *Mycobacterium tuberculosis* H_37_RV strain, with the lowest binding energy of −9.3 kcal/mol (Scheme 2).^43^

Krishnan et al. evaluated Biginelli pyrimidine products **14** and **16** for their anticancer activity against the A549, HT29, and HepG2 cell lines. By reacting urea **11** and ethyl acetoacetate **2** with two different aromatic aldehydes, namely 4-chlorobenzaldehyde **13** and 4-methoxybenzaldehyde **15**, to yield the respective dihydropyrimidines **14** and **16**. For these reactions, several optimization conditions were tested and screened with different catalysts, such as *p*-TSA, CAN, oxalic acid, and Cu­(OAc)_2_, which produced low to moderate yields, whereas TBAB resulted in the Knoevenagel condensed product. Finally, the best yields were obtained using imidazole hydrochloride as the efficient catalyst for this particular reaction. The dihydropyrimidine products were effective as anticancer molecules, showing a close resemblance to the standard drug doxorubicin (Scheme 3).^44^

Jadhav and coworkers provided rapid access to the Biginelli reaction to construct 1,2,3,4-tetrahydropyrimidines **23** at room temperature via the use of diisopropyl ethylammonium acetate **19** as an efficient ionic liquid catalyst. In addition to this ionic liquid catalyst, several attempts have been made with different catalysts, such as *p*-TSA, sodium dodecyl sulfate (SDS), and poly­(ethylene glycol) 400, which are moderate in action, whereas the utility of phase transfer catalysts (PTCs) is sluggish. When reacted with ethyl-2-cyanoacetate **21** and amines **22,** aromatic aldehyde **20** furnished final products **23** in excellent yields of up to 98% (Scheme 4).^45^

### 2.2 Metal-Catalyzed Reactions

In recent years, metal-catalyzed reactions have paved many routes for the efficient synthesis of interesting heterocyclic compounds. Among them, several works on pyrimidine have dragged greater attention for successfully executing complicated and complex reactions. Organic catalysis using Zn/Pd and Cu catalysts has left an indelible mark in the field of synthetic chemistry. Their role in accelerating the reaction has been significant, with various reaction optimizations. Here, we discuss the Zn-, Pd-, and Cu-catalyzed reactions, which yield many important pyrimidine core molecules.

#### 2.2.1 Zinc/Palladium-Catalyzed Reactions

An amino acid complex linked with zinc metal triggered the synthesis of novel pyrimidines **28** in an aqueous medium. The Zn­(l-proline)_2_ catalyst was prepared by mixing l-proline and triethylamine in ethanol and stirring for 20 min at room temperature. Furthermore, Zn­(AcO)_2_·6H_2_O was added dropwise to a small amount of water with continuous stirring at RT for 5–7 h. The white Zn-linked amino acid complex was filtered and dried under a vacuum for 4 h and employed as an efficient catalyst for the synthesis of pyrimidines from guanidines **27**. For the synthesis of the precursor, aromatic aldehydes **24** were reacted with acetophenones **25** in a mixture of water and ethanol to yield chalcone intermediates **26**, and their treatment with different benzoguanidines **27** led to the formation of target pyrimidines **28** (80–97% yield) facilitated by the Zn-complex catalyst in an aqueous medium (Scheme 5).^46^

The dehydrogenative synthesis of 2,4,6-triphenyl pyrimidines **32** was developed using Pd@Pt core–shell nanoparticles capped by polyvinylpyrrolidone (PVP) and supported by carbon; all of these nanoparticles were maintained and prepared in a flow reactor as a continuous process. This catalyst accelerated the reaction between primary **30**, secondary alcohols **29**, and amidines **31** in potassium *tert*-butoxide, which is the optimized base for the reaction. This synthesis is a part of a cascade of reactions involving the oxidation of alcohols to aldehydes and ketones and their cross-aldol condensation to give α, β-unsaturated ketone intermediates, whose reaction with amidines leads to the formation of pyrimidines through the C–N coupling reaction pathway in isolated yields of 65–85% (Scheme 6).^47^

Palladium-catalyzed isocyanide **34** insertion into 2*H*-azirines **33** led to the formation of polysubstituted pyrimidines **35** via the formation of one C–C and two C–N bonds with promising functional group tolerance. Palladium acetate and triphenylphosphine govern the reaction mechanism involving four main steps commencing with the oxidative addition of the azirine to the Pd complex, leading to the opening of the three-membered ring, followed by the isocyanide and repeating azirine insertions, which further led to ring expansion. Later, the reductive elimination and hydrolysis yield the target compounds with yields ranging from 0 to 98% (Scheme 7).^48^

#### 2.2.2 Copper-Catalyzed Synthesis

Tiwari et al. synthesized pyrazolopyrimidine-linked triazole glycohybrids **41** starting from diverse β-keto esters **36** obtained by the esterification of different acetophenones using diethyl carbonate. The presence of electron-withdrawing groups such as -trifluoromethyl and -fluoro in the aryl ring contributed to a lower yield, whereas electron-donating groups such as -methyl and -methoxy favored good yields. Pyrazolopyrimidine **38** was first obtained through a reflux reaction between β-keto esters **36** and 3-amino pyrazole **37** in acetic acid for 12–14 hours, yielding satisfactory results. The corresponding alkyne-pyrazolopyrimidines **39** were synthesized using propargyl bromide to facilitate an upcoming click reaction with azido glucoside **40** using copper sulfate/sodium ascorbate, resulting in their respective triazole-linked glycohybrids **41** in very high yields under microwave conditions (Scheme 8).^49^

Wang et al. annulated α, β-unsaturated ketoximes in a [4+2] fashion with activated nitriles to construct different 2,4,6-trisubstituted pyrimidines **44**. The reaction of chalcone-derived oxime acetates **42** with malononitrile **43**, catalyzed by Cu­(MeCN)_4_PF_6_ in dimethylacetamide (DMA) and 1,4-dioxane at a 1:9 ratio at 110 °C for 12 h, afforded the target compounds in an average yield of 82%. Several reaction conditions were examined, and after thorough investigation, the above reaction conditions were determined to be the most favorable for achieving promising yields. Apart from such optimizations, three different radical inhibitors, namely TEMPO, BHT, and DPE were utilized to check the performance of the reaction and produced compounds at 76–78% yields, whereas without them, the yield was slightly better (80%) (Scheme 9).^50^

Zarren et al. synthesized anthraquinone-based pyrimidine analogues **49** for the development of potent probes with antioxidant activity. The precursors anthraquinone **47** and pyrimidine derivatives **48** were synthesized in two separate parts. First, phthalic anhydride **45** was reacted with substituted benzenes **46** in the presence of alum at room temperature, followed by treatment with concentrated HCl to yield the precursor anthraquinone **47**. Second, substituted aromatic aldehydes **24** were transformed into pyrimidines **48** via their reactions with urea **11** and ethyl acetoacetate **2**. These two substrates were reacted in the presence of copper chloride, cupric oxide, and methanol to yield four different anthraquinone-pyrimidine probes **49** (Scheme 10).^51^

Putta et al. developed a copper-catalyzed dual synthon approach for the synthesis of pyrimidines **52** via the difunctionalization of tertiary alkylamines. In this method, tertiary alkylamines such as *N,N,N′,N*′-tetraethylethylenediamine (TEEDA) **51** act as efficient dual C2 synthons. In this two-component synthesis of pyrimidine, amidine hydrochloride **50** was reacted with TEEDA **51** in the presence of CuCl_2_ at 90 °C under aerobic conditions for 48 h. Various experiments were carried out to select superior catalysts, and finally CuCl_2_ exhibited the best performance. 1,4-Dioxane serves as the ideal solvent for the reaction, as DMSO, a one-carbon donor in several organic reactions, could not afford products under the above reaction conditions (Scheme 11).^52^

Li et al. constructed (*E*)-2,4-diaryl-6-styrylpyrimidines **55** from distyrylketones **53** and benzamidines **54** through a CuBr-catalyzed reaction in the presence of 2,2′-bypridine and DMSO at 100 °C for 12 h in an oil bath. The advantages of this reaction include the use of low-cost catalysts, easily available substrates, wide functional group tolerance, high atom economy, and the resulting products in yields of up to 98% (Scheme 12).^53^

### 2.3 Microwave/Ultrasound-Assisted Synthesis

In recent years, microwave and ultrasound techniques have been widely used due to their remarkable properties and the execution of reactions with several merits. Thus, their principles are a must-learn aspect for every organic chemist who is on the verge of excelling in the field of synthetic organic chemistry. The major advantages of these techniques include reduced time consumption, high-yield products, minimal use of solvents, etc. These merits have enabled various researchers to undertake pyrimidine chemistry under microwave/ultrasound principles, as discussed below.

Trivedi et al. synthesized pyrimido­[4,5-*d*]­pyrimidines **59** from barbituric acid **56**, aromatic aldehydes **57**, and amines **22** under microwave conditions for 5 min in the presence of an iodine solution prepared by dissolving iodine in potassium iodide. An additional 50 mL of water was added before the reaction was placed into a microwave reactor at 640 W. The same reaction was carried out under ultrasound, and it was found that the reaction duration was longer with a lower yield compared to microwave irradiation (Scheme 13).^54^

Panneerselvam and Mandhadi constructed thiosemicarbazide derivatives of pyrimidine **69** via a microwave synthesizer from the substrate aromatic aldehydes **60**, ethyl-2-cyanoacetate **21**, and guanidine hydrochloride **61** to yield the intermediate 2-amino-4-hydroxy-6-(substituted benzyl)­pyrimidine-5-carboxamide **62**. This intermediate **62** was then esterified using ethyl-2-bromoacetate **63** and propanone **64** to give ethyl-2-((2-amino-5-carbamoyl-6-(substituted benzyl) pyrimidin-4-yl)­oxy)­acetate **65**, whose condensation with thiosemicarbazide **66** led to the formation of prefinal compounds 2-amino-4-(2-[2-carbamothioylhydrazinyl]-2-oxoethoxy)-6-(substituted benzyl)­pyrimidine-5-carboxamides **67**. In the final step, it was treated with acetaldehyde **68** to give the final compounds **69**. All the reactions were carried out under microwave irradiation and furnished products in 3–5 min with a yield of 63–82% (Scheme 14).^55^

Alizadeh et al. designed pyrrole-fused pyrimidine **75** via an ultrasound method involving the reaction between arylglyoxals **70** and malononitrile **43** to yield 2-(2-oxo-2-arylethylidene) malononitrile **71** as the first precursor for the final reaction. On the other hand, the second precursors, viz., ketene aminals **74**, were prepared from 1,1-bis­(methylthio)-2-nitroethylene **72** and diamines **73** with 20 kHz ultrasonic radiation. The precursors were then combined to give pyrrole-fused pyrimidine analogues **75** with 39–95% (Scheme 15).^56^

Kerru et al. employed an ultrasound-assisted synthetic protocol for the synthesis of benzothiazole­[3,2-*a*]­pyrimidine derivatives **78** via MCR involving 2-aminobenzothiazole **76**, aromatic aldehydes **57**, and substituted nitriles **77** in the presence of fused ammonia and ethanol at room temperature. The most fascinating factor is their extremely well-established yields in all the cases of the reactions, which are above 94% (Scheme 16).^57^

Thavasianandam Seenivasan et al. investigated the substrate scope of CF_3_–ynones **79** for the synthesis of polyfluoro-pyrimido [1,2-*a*]­benzimidazole analogues **81** via an ultrasound-assisted technique. First, various classes of CF_3_–ynones **79** were treated with 2-aminobenzimidazole **80** and reacted in an ultrasonicator in a neat, open-air atmosphere for an hour to yield the final compounds **81**. This reaction was facilitated without the use of solvents or metals and produced compounds with high functional group tolerance, with yields ranging from 28 to 95% (Scheme 17).^58^

## 3 Functionalization of Pyrimidines

Interestingly, several attempts have been made to modify the pyrimidine nucleus with different functional groups in recent years. By making such modifications, many doors could open for the production of medicinally important pyrimidine-containing scaffolds. Functionalization can be achieved by replacing one hydrogen atom from the pyrimidine core with a different functional group or substituent. Thus, we discuss recent developments in the functionalization of pyrimidine heterocycles.

Das et al. carried out remote C–H functionalization of 2-aminopyrimidines **82** via a palladium-catalyzed reaction. Arylation of 2-aminopyrimidine core **82** with aryl halides **83** emerged as a highly regioselective approach. Moreover, it displayed good tolerance to different aryl halides and produced functionalized products **84** in satisfactory yields (Scheme 18).^59^

Zhang et al. developed a unique regioselective approach enabling palladium-catalyzed, sodium iodide-promoted C–H diacetoxylation of pyrrolo­[2,3-*d*]­pyrimidine derivatives **85**. This diacetoxylation was performed exclusively at the C-4 phenyl ring and the C-5 position of the pyrrole ring. Various electron-donating groups afforded products **86** in good yields (Scheme 19).^60^

Muzychka et al. synthesized triphenylphosphonium-functionalized pyrimidines **90–91** for antibiofilm activity. Methyl [3,5-dibromo-4-(2-bromomethoxy)­phenyl]­acetate **87** was treated with phosphoranylidenepyrimidines **88** and **89** to yield the target triphenylphosphonium-containing pyrimidine derivatives **90** and **91** under reflux with acetonitrile (Scheme 20).^61^

Pilli et al. functionalized 2,4-dichloropyrimidine intermediate **97** synthesized from the Wittig reaction/hydrogenation procedure of salicylaldehyde **92,** yielding tertiary butyl ester **94**, whose S_N_2 reaction with bromopropanol **95** was followed by a Mitsunobu reaction involving diisopropyl azodicarboxylate (DIAD), triphenylphosphine, and 5-hydroxy-2,4-dicholoropyrimidine **97**. Later, Negishi and Sonogashira reactions were performed on this 2,4-dichloropyrimidine to obtain highly functionalized products (Scheme 21).^62^

## 4 Biology of Pyrimidines

Pyrimidine is a biologically important and versatile heterocyclic compound. By experimenting with several reactions involving pyrimidine molecules, numerous drugs are now commercially available, which help in curing various deadly diseases and inhibit transmissions from pathogens. The medicinal importance of pyrimidines has been discussed in this section, which aims to provide new insights into the domain of pharmaceutical chemistry.

### 4.1 Antimicrobial Activity

Antimicrobial activity includes antibacterial^63^ and antifungal^64^ effects. Thus, these activities help pharmacists and chemists understand the chemical and medicinal effects of treating infections.^65^ Several drug molecules are available in the commercial sector, such as trimethoprim,^66^ cyprodinil,^67^ and sulfadiazine,^68^ as shown in Table 1.

Ibrahim et al. produced novel heteroannulated chromene-pyrido-thiazolo-pyrimidines from an aldehyde derivative in DMF/DBU and tested their antibacterial activity against Gram-positive (*Staphylococcus aureus* and *Bacillus subtilis*) and Gram-negative (*Salmonella typhimurium* and *Escherichia coli*) strains. Compared with chloramphenicol, cycloheximide, and cephalothin, which are standard reference drugs, compounds **101–103** showed prominent activity against both Gram-positive and Gram-negative bacteria. The inhibition values were 1000 and 500 μg/mL, respectively. Compounds **104–105** showed high antibacterial activity against Gram-positive bacteria only. They also investigated the compounds for their antifungal activity against *Aspergillus fumigatus* and reported that compounds **99–106** displayed high activity. Therefore, heteroannulation profoundly increased the antibacterial and antifungal activity (Scheme 22).^69^

Alamshany and Nossier synthesized new thiazole-linked pyrimidine derivatives and investigated their antimicrobial activity against *Staphylococcus aureus*, *Streptococcus faecalis*, *Escherichia coli*, *Klebsiella pneumoniae*, *Saccharomyces cerevisiae*, and *Candida albicans*. Except for *C. albicans*, compounds **108/109** effectively inhibited all of the bacteria and yeast. The minimal inhibitory concentrations (MICs) of compounds **108/109** were 0.20 ± 0.02/0.25 ± 0.01 (*S. aureus*), 0.38 ± 0.06/0.45 ± 0.01 (*S. faecalis*), 0.49 ± 0.03/0.44 ± 0.05 (*E. coli*), 0.41 ± 0.05/0.45 ± 0.03 (*K. pneumoniae*), and 0.32 ± 0.04/0.42 ± 0.06 (*S. cerevisiae*). The zones of inhibition were measured (in mm) for compounds **108/109** against all the microbial species and were reported to be 30 ± 0.75/29 ± 0.81 (*S. aureus*), 28 ± 0.47/25 ± 0.14 (*S. faecalis*), 25 ± 0.37/23 ± 0.18 (*E. coli*), 26 ± 0.18/25 ± 0.65 (*K. pneumoniae*), and 28 ± 0.29/26 ± 0.40 (*S. cerevisiae*), respectively. Chloramphenicol and ketoconazole were used as the standard drugs for evaluation (Scheme 23).^70^

Badiger and Kamanna developed a greener approach for synthesizing pyranopyrimidine derivatives via the use of the eco-friendly catalyst water extract of pomegranate peel ash (WEPPA) under microwave conditions and subjected this approach to antimicrobial studies against *Bacillus*, *E. coli*, *Pseudomonas*, *Candida*, and *Aspergillus*. Ciprofloxacin and fluconazole were used as reference standards for the activity. Among the synthesized hybrids, compounds **111–114** showed good antibacterial and antifungal activity at different concentrations. All the compounds **111–114** showed inhibition of 10–28 mm at 75 μL/mL (Scheme 24)^71^ (Figure 3).

### 4.2 Antitubercular Activity

*Mycobacterium tuberculosis* is a serious threat to the lungs of infected individuals who severely cough or sneeze. This transmission is risky and fatal. To treat this condition, the FDA has already approved several drugs that are economically available, such as isoniazid, pyrazinamide, ethambutol, and rifampicin. Since these antibiotics are often inadequate, there is a great requirement to develop effective medicinal hybrids that are active against disease-causing *Mycobacterium* pathogens.

Sun et al. synthesized pyrimidine derivatives and tested their antitubercular activity against the *Mycobacterium tuberculosis* H37Rv strain. Compounds **117** and **118** exhibited the highest MICs (μg/mL) of 0.16 and 0.12, respectively. Additionally, they achieved chiral resolution of these two compounds to form *R*-forms **117a**/**118a** and *S*-forms **117b**/**118b**. By doing so, **117a**/**118a** exhibited significant antitubercular activity with an MIC (μg/mL) of 0.03–0.06 against the H37Rv strain, along with low hERG toxicity. Furthermore, it was revealed that these compounds possessed good metabolic stability. The in vivo activity demonstrated that the larvae and adults of zebrafish infected with *Mycobacterium marinum* displayed good therapeutic action (Scheme 25).^72^

Li and coworkers constructed novel pyrimidine hybrids and evaluated their ability to inhibit tuberculosis. The compounds were tested against *Mycobacterium tuberculosis* H37Ra and H37Rv strains. These compounds were also compared with the clinical drug-resistant TB. Compound **125** was identified as the lead hybrid, exhibiting remarkable potency in inhibiting strains with MIC values (μg/mL) of 0.5–1.0. Thus, compound **125** shows promise as a prime compound for treating drug-resistant TB. The SAR studies demonstrated that replacing the naphthyl group with hydrophobic substitutes, such as a phenyl ring in LPX-16j, resulted in good tolerance. LPX-16j is the potential drug candidate as an antitubercular agent. However, the presence of the core pyrimidine nucleus remains crucial for displaying significant antitubercular action in compound **125** (Scheme 26).^73^

Hemeda et al. produced pyrimidine-linked benzothiazole analogues and investigated their antitubercular activity against various tuberculosis strains. After careful evaluation, compounds **130, 131, 133,** and **134** showed high activity against *M. tuberculosis* (ATCC 25177) with MIC values (μg/mL) of 0.24–0.98. Moreover, compounds **130** and **134** were very active against the resistant strain, with MIC values (μg/mL) of 0.98 and 1.95, respectively (Scheme 27)^74^ (Figure 4).

### 4.3 Antidiabetic Activity

Diabetes mellitus is one of the major conditions affecting people worldwide. To combat this condition, scientists are striving to develop effective drug molecules that can significantly reduce blood glucose levels.^75,76^ Various pyrimidine-containing drugs are commercially available, namely gemigliptin,^77^ linagliptin,^78^ gosogliptin,^79^ and anagliptin^80^ as listed in Table 2.

Amin et al. synthesized thiazolidinedione-linked pyrimidine derivatives and conducted in silico studies along with biological evaluation of their antidiabetic performance. To improve hyperglycemia-related complications in diabetic people, such derivatives were constructed and studied. ADME studies revealed that compounds **141** and **142** were within the range of Lipinski’s rule of five, a well-known guideline in pharmacology. These compounds showed notable performance in the oral glucose tolerance test (OGTT) and were further subjected to an antidiabetic test in streptozotocin-induced diabetic rats for 4 weeks. It significantly reduced the blood glucose levels to 145.2 ± 1.35 and 146.6 ± 0.81, respectively. These results were even stronger than the results obtained from the standard drug pioglitazone (150.2 ± 1.06). The SAR studies revealed the importance of the thiazolidinedione moiety of compounds **141** and **142** at he position *ortho* to the phenoxy ring, displaying better activity than those present at the *meta* position. Moreover, the methoxy group at the para position in compound **141** showed better activity than compound **142** having fluorine at the same position. The isopropyl side chain linked to the sulfur atom in compounds **141–142** had greater activity than the *n*-propyl side chain, thus revealing the effect of the alkyl side chain on antidiabetic activity (Scheme 28).^81^

Mallidi and coworkers produced pyrimidine-tethered carbocyclic nucleoside analogues and performed in silico antidiabetic evaluations. After thorough investigation, compounds **151** and **154** showed noteworthy results against α-glucosidase with IC_50_ values (nmol) of 43.292 and 48.638, respectively. The *E*-isomers of both compounds were selectively promising because of their outstanding results (Scheme 29).^82^

Toan and colleagues synthesized novel pyrimidine–coumarin hybrids and studied their effectiveness toward antidiabetic properties by testing them against two well-known enzymes, viz. α-glucosidase and α-amylase, which hydrolyze glycoside linkages. Acarbose was used as the standard drug for the experiment. Compound **158** was effective against α-amylase with an IC_50_ value (μM) 102.32 ± 1.15, whereas compound **159** displayed an IC_50_ value of 115.82 ± 1.12. The reason for this slight decrease in inhibition is the change in the chloro substituent from the 3rd position to the 4th position. Compound **160** was most effective against α-glucosidase, with an IC_50_ value (μM) of 52.16 ± 1.12. Overall, compounds **161**, **158**, and **160** showed good activity with IC_50_ values (μM) of 82.6 ± 1.15, 96.64 ± 1.15, and 98.53 ± 1.17, respectively. Therefore, it was clear from this study that the compounds were more effective in inhibiting α-glucosidase than in inhibiting α-amylase. The SAR studies highlighted the phenomenal substitution of the phenyl ring with the chloro group in compounds **158** and **159** showed incredible inhibitory activity against α-amylase, whereas bromo groups decreased the activity. The presence of the strong electron-donating methyl and methoxy groups in compounds **160** and **161** contributed significantly to inhibiting α-glucosidase activity and decreasing α-amylase activity (Scheme 30)^83^ (Figure 5).

### 4.4 Comparison of Biologically Active Compounds

The overall comparison of the biologically active compounds discussed is listed in Table 3. With this comparative description, it is easy to locate the compounds and their actions as discussed in the above section.

## 5 Conclusion

The latest progress and advances in the synthetic development of pyrimidines have had a consequential impact on the rapidly growing pharmaceutical and academic sectors. The synthetic strategies discussed here involve recent modifications and improvements in the reactions and conditions that produce lead compounds. The biological impact of pyrimidine derivatives opens a wide gateway for emerging drug development research. Therefore, the wide range of implications and scope of pyrimidines are at the heart of heterocyclic chemistry, which has led to the performance of high-class research in the development of biologically active and potent molecules. Several of the compounds discussed in this review are produced in excellent yields with different reaction optimizations and modifications. Compared with the traditional methods, the use of microwave/ultrasound-promoted synthetic methods results in lead molecules in high yields with greater purity. Direct functionalization of pyrimidines was noteworthy in providing the final compounds with favorable outcomes. A few pyrimidine-containing hybrids were even better than the standard reference drug at acting against pathogens/microorganisms. Therefore, the construction of new pyrimidine-containing analogues is necessary for the development of new biologically well-performing molecules, as infections, transmission, and other ailments have put human life at risk.

## Data Availability

All the data were obtained from peer-reviewed articles cited in the reference list, with no additional data sets utilized.
